# CircCENPM serves as a CeRNA to aggravate nasopharyngeal carcinoma metastasis and stemness via enhancing BMI1

**DOI:** 10.1186/s41065-025-00406-7

**Published:** 2025-03-14

**Authors:** Rui Wang, Fei Wang

**Affiliations:** 1 The Second Department of Otolaryngology-Head and Neck Surgery, The First Affiliated Hospital of Kunming Medical University, Kunming, Yunnan, 650032 China; 2 Department of Ultrasound, The First Affiliated Hospital of Kunming Medical University, No. 295 Xichang Road, Wuhua District, Kunming, Yunnan, 650032 China

**Keywords:** NPC, CircCENPM, miR-362-3p, BMI1, Metastasis

## Abstract

**Background:**

Nasopharyngeal carcinoma (NPC) is a malignant head and neck cancer with high mortality and dismal prognosis. Emerging research have disclosed that circRNAs are crucial gene expression regulators engaged in tumor advancement. This work aspired to identify novel oncogenic circRNA driving NPC progression.

**Methods:**

Bioinformatics analysis was performed to explore and predict underlying circRNA and downstream targets. Luciferase reporter assay was executed to check the binding relationship between these genes. Cell function tests were conducted using CCK-8, would healing, and flow cytometry. The stemness markers CD133, Nanog and Oct4 was detected via western blot.

**Results:**

CircCENPM was notably enhanced in NPC. Silencing of circCENPM suppressed NPC cell growth, migration, and stemness in vitro, simultaneously impeded tumorigenesis of NPC in vivo. Moreover, circCENPM could interact with miR-362-3p, whereas miR-362-3p inhibitor apparently reversed the mitigated growth and stemness induced by circCENPM knockdown in NPC cells. Furthermore, BMI1 was identified to be the downstream target of miR-362-3p, and BMI1 introduction partially offset the anti-tumor function of miR-362-3p in NPC cells.

**Conclusion:**

CircCENPM functioned as a carcinogenic driver and facilitated NPC growth and stemness via miR-362-3p/BMI1 regulatory network, which provided a potential biomarker and attractive target for NPC intervention and treatment.

**Supplementary Information:**

The online version contains supplementary material available at 10.1186/s41065-025-00406-7.

## Introduction

Nasopharyngeal carcinoma (NPC) is a malignancy originating from nasopharyngeal epithelial cells, generally found in the top and lateral walls of the nasopharynx [1]. The World Health Organization classifies NPC into three subtypes: keratinizing squamous cell carcinoma, nonkeratinizing differentiated carcinoma, and nonkeratinizing undifferentiated carcinoma [1]. Notably, the incidence of NPC has significant regional characteristics, mainly distributed in Southeast Asia and North Africa [2]. Epidemiologic studies have disclosed that the advancement of NPC is the result of the interaction of multiple factors, such as Epstein-Barr (EB) virus infection, environmental, and genetic factors [3]. The location of NPC is insidious, and patients usually lack specific clinical manifestations in the early stage, most of them are diagnosed in the middle-late stage, accompanied by symptoms of distant metastasis [4]. Currently, radiation therapy is an effective strategy for early-stage NPC patients, while middle-late-stage patients are mostly managed with the integrated treatment of radiotherapy combined with drugs [5]. Although NPC treatment has achieved certain efficacy, the five-year survival rate of NPC remains unsatisfactory, in which local recurrence and distant metastasis are the principal causes for treatment failure [6]. Therefore, further research on the molecular mechanisms affecting NPC metastasis/relapse and searching for appropriate early diagnostic biomarkers are of vital significance for the precise treatment and prognostic assessment of NPC.

CircRNAs are a class of non-coding RNAs (nc RNAs) with a covalent ring structure, widely found in eukaryotic organisms [7]. They are formed by back-splicing of pre-mRNAs, and without a 5′-cap or a 3′-poly(A) tail [8]. In particular, circRNA is not susceptible to be degraded, and it can stably exist in body fluids with high tissue specificity [9]. Recent years have witnessed a spurt of progress in sequencing and bioinformatics, circRNAs are no longer regarded as a product of splicing errors. To date, circRNAs have been validated to possess abundant biological functions, including serving as microRNA (miRNA) sponges, modulating gene transcription, interacting with RNA-binding proteins, and translated proteins [10]. Importantly, a growing number of reports uncovered that circRNAs function as key regulatory factors in the advancement of malignancy by affecting the differentiation, proliferation, distant metastasis, and apoptosis processes of tumor cells [11]. Additionally, the abundant expression of circRNAs in tumor tissues and plasma has specificity and stability, indicating that circRNA endows promising clinical application value in disease diagnosis and treatment [12].

In NPC, Liang et al. constructed nine-circRNA signature for prognosis and treatment decisions [13]. Hong et al. identified circIPO7 as a prognostic biomarker after cisplatin-based chemotherapy [14]. In addition, it is worth noting that the presence of NPC stemness is an important factor driving tumor metastasis and relapse [15, 16]. Exosomes loaded with circPARD3 have been evidenced to induce NPC stemness and augment tumor metastasis [17]. However, extensive circRNAs and their specifc regulatory mechanisms in the stemness and metastasis/relapse of NPC deserve further exploitation.

Here, our current work identified circCENPM as a critical tumour promoting circRNA that endows NPC cells with stemness phenotype and metastatic ability. CircCENPM was substantially elevated in NPC, silencing of circCENPM suppressed NPC metastasis and stemness. Additionally, we revealed the mechanism by which circCENPM performs as ceRNA to modulate NPC progression. In sum, our findings confirmed the essential role of circCENPM in driving NPC tumorigenesis, which may contribute to refresh the targets and insights into the treatment of NPC.

## Materials and methods

### Bioinformatics analysis

GEO online dataset GSE190271 (https://www.ncbi.nlm.nih.gov/geo/) was employed to identify the circRNA with metastatic/relapse signature in NPC patients. The differentially upregulated circRNAs from tissues (NPC patients vs. healthy control; NPC patients with posttreatment relapse vs. NPC patients without posttreatment relapse) in GSE190271 dataset were intersected, and 4 potential circRNAs were obtained. Circbank (http://www.circbank.cn/) and ENCORI (https://rnasysu.com/encori/) databases were utilized to predict underlying downstream miRNAs of circCENPM. Also, ENCORI, miRmap (https://mirmap.ezlab.org/app), and GSE227541 were searched for potential downstream mRNAs. Additionally, LinkedOmics database (https://www.linkedomics.org/login.php) was used to conduct enrichment analysis of co-expression genes associated with BMI1 in Head-and-neck Carcinoma.

### Tissue samples

Thirty pairs of NPC tissue samples and their corresponding adjacent tissues were collected from the First Affiliated Hospital of Kunming Medical University. All these samples were confirmed through historical examination. This research was authorized by the Ethics Committee of the First Affiliated Hospital of Kunming Medical University and received written consent from patients. After the tissue specimens were taken out, they were promptly frozen in liquid nitrogen and then stored at -80℃ for subsequent experiments. Besides, the NPC patients information was presented in Supplementary Table 1.

### Cell culture

The immortalized normal human nasopharyngeal epithelial cell line (NP69) and NPC cell lines (5–8 F, HNE2, CNE2, HONE1, and 6-10B) were bought from American Type Culture Collection. NP69 cells were grown with keratinocyte/serum-free medium (Gibco, USA). NPC cells were cultured in RPMI 1640 (Gibco) carrying 10% FBS. The above cells were maintained at 37℃ chamber in 5% CO_2_.

### Cell transfection

CircCENPM small interfering RNA (si-circCENPM#1/si-circCENPM#2), siRNA negative control (si-NC), miR-362-3p mimics/miR-362-3p inhibitors and the negative controls (mimics NC/inhibitors NC), BMI1 overexpression vector (pcDNA-BMI1) and pcDNA empty vector were synthesized by GenePharma (Shanghai, China). NPC cells (HNE2, CNE2) were inoculated in 6-well plates. When cells grown to 80% confluence, the above oligonucleotides and vectors were transfected into HNE2 and CNE2 cells via Lipofectamine 3000 (Invitrogen, USA). Human lentivirus-sh-circ_CENPM and negative control lentivirus were packed and purchased from GenePharma. NPC cells were infected with these lentiviral particles based on manufacturer’s protocol.

### qRTPCR

Total RNA was extracted from gathered tumor samples and cultured cells by employing TRIzol reagent (Invitrogen). RNA (2 µg) was converted into cDNA through PrimeScript RT Master Mix (Takara, Japan). Next, qRT-PCR was accomplished with SYBR Premix Ex Taq (Takara, Japan) on ABI 7500 real-time PCR system. GAPDH and U6 were applied as internal reference and the relative expression of target genes was calculated through 2^−∆∆Ct^ methods. Primer sequences were displayed as below (5′→3′):

Circ_CENPM-F: TGCTCTACTGTGACCTGGAG.

Circ_CENPM-R: CAGCAAGATGGTGGCCGTGTTCAGCC.

miR-362-3p-F: AACACACCTATTCAAGGATTCA.

miR-362-3p-R: ACGTGACACGTTCGGAGAATT.

BMI1-F: TCATCCTTCTGCTGATGCTG.

BMI1-R: GCATCACAGTCATTGCTGCT.

GAPDH-F: TATGATGATATCAAGAGGGTAGT.

GAPDH-R: TGTATCCAAACTCATTGTCATAC.

U6-F: CTCGCTTCGGCAGCACA.

U6-R: AACGCTTCACGAATTTGCGT.

### Treatment of RNase R

Total RNA samples (2 µg) from HNE2 and CNE2 cells were treated with 3 U/mg RNase R (ab286929, Abcam) for 30 min at 37 °C. Later on, circCENPM and linear CENPM expression were measured through qRTPCR.

### Subcellular location assay

In subcellular localization analysis, PARIS Kit (Invitrogen) was applied to separate nuclear and cytoplasmic RNAs from NPC cells depending on the protocol suggested. Subsequently, the abundance of circ_CENPM distributed in nuclear and cytoplasm was quantified by employing qRT-PCR, with U6 and GAPDH as the internal reference.

### CCK-8

NPC cells were inoculated into 96-well plates at a density of 2000 cells/well according to the experimental groups, each group included 5 wells, and cultured in an incubator. As suggested by CCK-8 kit’s protocol (Beyotime, China), 10 µL of CCK-8 reagent was added into 96-well plates and incubated at 37℃ for 2 h. Subsequently, the absorbance in each well was examined under a microplate reader at 450 nm wavelength for 4 consecutive days.

### Would healing assay

After digesting, NPC cells were resuspended in medium and seeded in 6-well plates at 5 × 10^5^ cells/well. When cell density reached 90%, a sterile 200 µL pipette tip was utilized to draw a gap. Subsequently, these cells were maintained in serum-free medium for 24 h. The images of cells were taken through the microscope at 0, 24 h respectively. Finally, wound widths were examined with ImageJ.

### Flow cytometry

Annexin V-FITC/PI kit (Keygen Biotech, China) was utilized for cell apoptosis assessment. Briefly, NPC cells were harvested and re-suspended in the flow tube, followed by treating with 5 µl Annexin V-FITC and 5 µL PI. Finally, the cell apoptosis was examined via a flow cytometer.

### Western blot

Proteins from transfected NPC cells were extracted with RIPA lysis buffer (Beyotime, China). BCA kit (Sigma, USA) was employed to quantified total proteins. Next, equal amounts of protein samples (30 µg per lane) were separated through SDS/PAGE, and then transferred onto PVDF membranes (Millipore, USA). After blocking in 5% skimmed milk at room temperature for 1 h, the membranes were incubated with primary antibodies overnight at 4℃, including anti-CD133, anti-Nanog, anti-Otc4 and anti-GAPDH antibodies (Abcam). Afterwards, these membranes were immersed with secondary antibody at room temperature for 1 h. Finally, ECL kit was utilized to display protein bands.

### Dual-Luciferase reporter assay

Dual-Luciferase Reporter gene detection was conducted to validate the relationships between miR-362-3p and circCENPM or BMI1. Wild-type (WT) or mutant-type (MUT) sequences of circCENPM or BMI1 3’UTR were cloned into the pmirGLO vector (Promega, Madison, WI, USA) to construct desired luciferase reporter vectors (WT-circCENPM, MUT-circCENPM, WT-BMI1 and MUT-BMI1). Afterward, NPC cells were co-transfected with these vectors and miR-362-3p mimics or mimics NC through Lipofectamine 3000. After 48 h, luciferase activities were measured using a dual luciferase assay system (Promega, USA).

### Animal models

The procedures of animal experiments were authorized by the First Affiliated Hospital of Kunming Medical University. Male BALB/c nude mice (6–8 weeks) were purchased from Vital River (Beijing, China) and randomly divided into 2 groups (*n* = 3): sh-NC group and sh-circCENPM group. 100 µL suspension of HNE2 cell stably silencing circCENPM or NC with a cell density of 2 × 10^7^/ml was subcutaneously injected into nude mice. The tumor volumes (length × width^2^ × 0.5) were recorded every 5 days using a vernier caliper. 25 days later, these mice were sacrificed, and tumor samples were resected, followed by photographing and weighing.

### Statistical analysis

The data gathered from three independent experiments were statistically analyzed through GraphPad Prism 8 (La Jolla, USA). Measurement data were expressed as mean ± standard deviation. The difference analysis between groups were calculated using Student’s t-test or ANOVA. The expression correlation between circCENPM, miR-362-3p, and BMI1 were analyzed through Pearson’s analysis. *P* < 0.05 indicated statistical significance.

## Results

### CircCENPM was notably enhanced in NPC

**Fig. 1:**
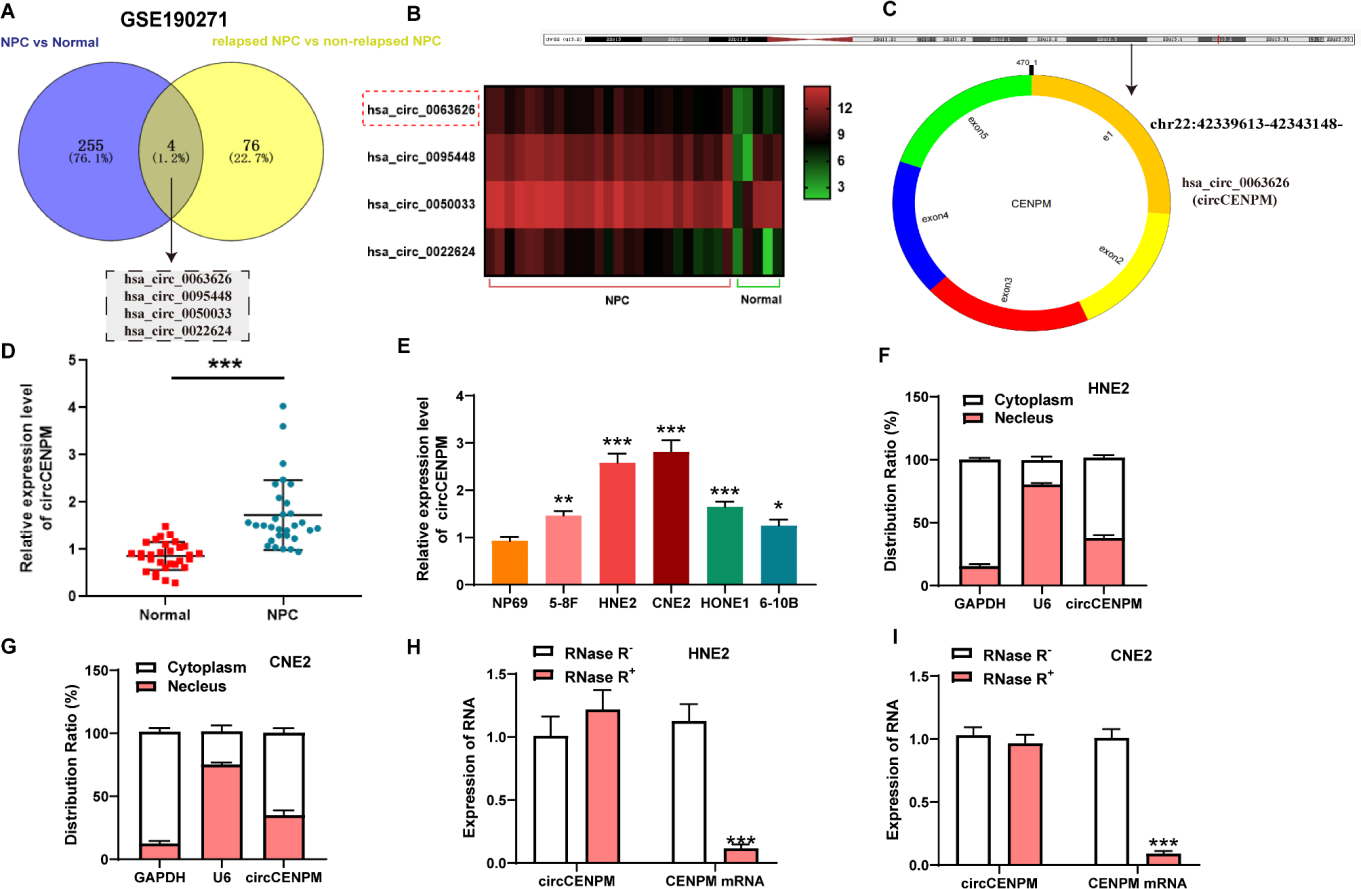
CircCENPM was notably enhanced in NPC. (**A**) The differentially upregulated circRNAs from NPC tissues was analyzed using GSE190271 dataset. (**B**) The heat maps of 4 different circRNA expression was generated using GSE190271 dataset. (**C**) Location and formation of circCENPM was revealed through circPrimer software. (**D**) qRT-PCR indicated that circCENPM was elevated in NPC tissues (*n* = 30). (**E**) qRT-PCR revealed that circCENPM was upregulated in NPC cells. (**F-G**) qRT-PCR was conducted to evaluate circCENPM abundance in cytoplasm and nucleus of NPC cells. (H-I) The stability of circCENPM was measured in NPC cells treated with RNase R using qRT-PCR. *P < 0.05, **P < 0.01, ***P < 0.001

Based on GSE190271 dataset, we identified the novel circRNA with metastatic/relapse signature in NPC patients. The differentially upregulated circRNAs from tissues (NPC patients vs. healthy control; NPC patients with posttreatment relapse vs. NPC patients without posttreatment relapse) in GSE190271 dataset were intersected, and 4 potential circRNAs were obtained (Fig. 1A). Then we evaluated the expression levels of these 4 circRNAs in NPC tissues and healthy control tissues by making heat maps (Fig. 1B). Among them, we chose hsa_circ_0063626 with obvious difference expression as our research object. The software circPrimer indicated that hsa_circ_0063626 (circCENPM) was generated by back splicing of exon 1 to exon 5 of CENPM gene (Fig. 1C). To characterize the expression pattern of circCENPM in NPC, we examined circCENPM levels in NPC tumor samples and paracancerous normal samples collected from 30 NPC patients. qRT-PCR showed that circCENPM was dramatically elevated in NPC samples (Fig. 1D). Also, circCENPM expression in patients with advanced NPC (III + IV) was markedly higher than that in the early NPC patients (I + II), and Kaplan-Meier survival analyses revealed that enhanced circCENPM level was indicative of worse outcomes (Fig. S1A-B). Similarly, we measured the circCENPM exprssion in NPC cells cultured in vitro. The results exhibited that circCENPM was substantially enhanced in NPC cells (Fig. 1E). Meanwhile, we selected two cell lines (HNE2 and CNE2) with relatively high expression in NPC as the basic cell lines for following studies. Before exploring the bio-function of circCENPM in NPC, we investigated the characteristics of circCENPM. Subcellular localization analysis disclosed that circCENPM was mainly located in the cytoplasm of NPC cells (Fig. 1F-G). Afterwards, RNase R digestion methods were conducted to identify the stability of circCENPM in NPC cells. We discovered that circCENPM was resistant to RNase R, its expression had no apparent change after RNase R treatment, while linear CENPM expression notably decreased (Fig. 1H-I). Collectively, circCENPM was substantially enhanced in NPC, suggesting it functions as a carcinogenic role in NPC advancement.

### CircCENPM knockdown restrained the growth, metastasis, and stemness of NPC

**Fig. 2:**
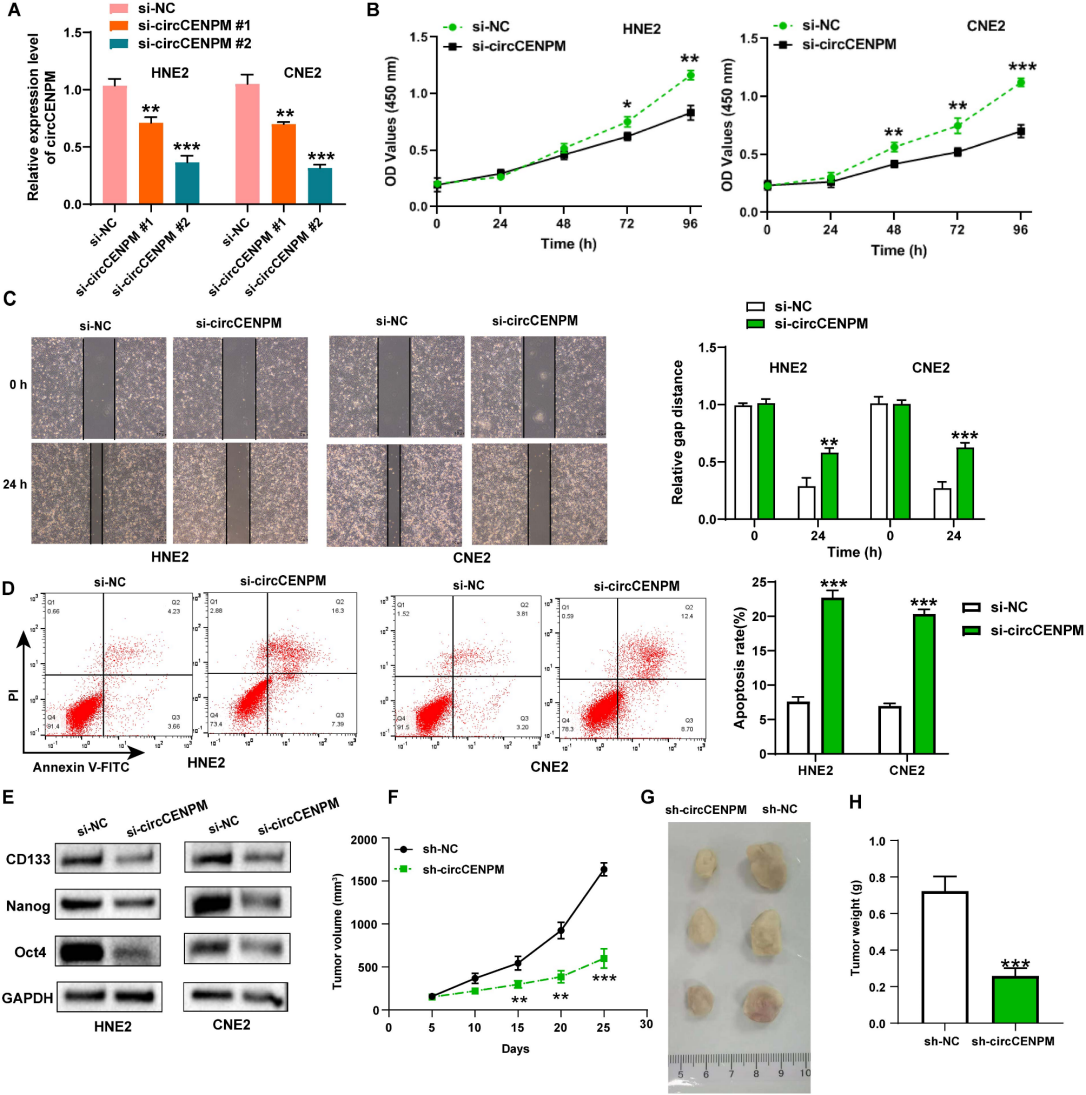
CircCENPM knockdown restrained the growth, metastasis, and stemness of NPC. (**A**) The inhibitory efficiency of siRNA on circCENPM was measured via qRT-PCR. (**B**) CCK-8 assay was conducted to assess NPC cell viability after circCENPM knockdown. (**C**) Wound healing assay was utilized to evaluate NPC cell migration after circCENPM knockdown. (**D**) Flow cytometry was executed to identify NPC cell apoptosis after circCENPM knockdown. (**E**) The stemness markers were detected in NPC cells after circCENPM knockdown. (**F**) Tumor volume of mice in sh-circCENPM and sh-NC was examined. (**G**) Photographs of tumor tissues from circCENPM silencing mice and control mice. (H) Tumor weight was shown. *P < 0.05, **P < 0.01, ***P < 0.001

To identify the critical role of circCENPM in the malignant development of NPC, we first constructed NPC cell lines with low expression of circCENPM. qRT-PCR presented that circCENPM was dramatically diminished in NPC cells transfected with si-circCENPM#1/#2 (Fig. 2A). Meanwhile, si-circCENPM#2 showed higher knockdown efficiency, so si-circCENPM#2 was selected for subsequent functional tests. CCK-8 detection showed that circCENPM downregulation potently mitigated the viability of NPC cells (Fig. 2B). Wound healing disclosed that circCENPM silencing obviously restrained the migratory capabilities of NPC cells (Fig. 2C). Also, flow cytometry analysis revealed that circCENPM silencing accelerated the apoptosis of NPC cells (Fig. 2D). Next, we evaluated the change of stemness markers expression in NPC cells through western blot. The outcomes revealed that the stemness markers, including CD133, Nanog, and Oct4, were declined after circCENPM downregulation (Fig. 2E). Furthermore, to validate whether circCENPM affects NPC tumorigenesis in vivo, we constructed animal models by injected HNE2 cells transfected with sh-NC and sh-circCENPM into nude mice. We recorded the tumor volume every 5 days and gathered tumor tissues from sacrificed nude mice after 25 days. The results exhibited that tumor volume was notably mitigated in sh-circCENPM group compared to sh-NC group (Fig. 2F). Moreover, circCENPM silencing remarkably diminished the size and weight of NPC tumor in mice (Fig. 2G-H). Taken together, circCENPM deficiency impeded tumorigenesis of NPC in vitro and in vivo.

### CircCENPM directly bound to miR-362-3p in NPC cells

**Fig. 3:**
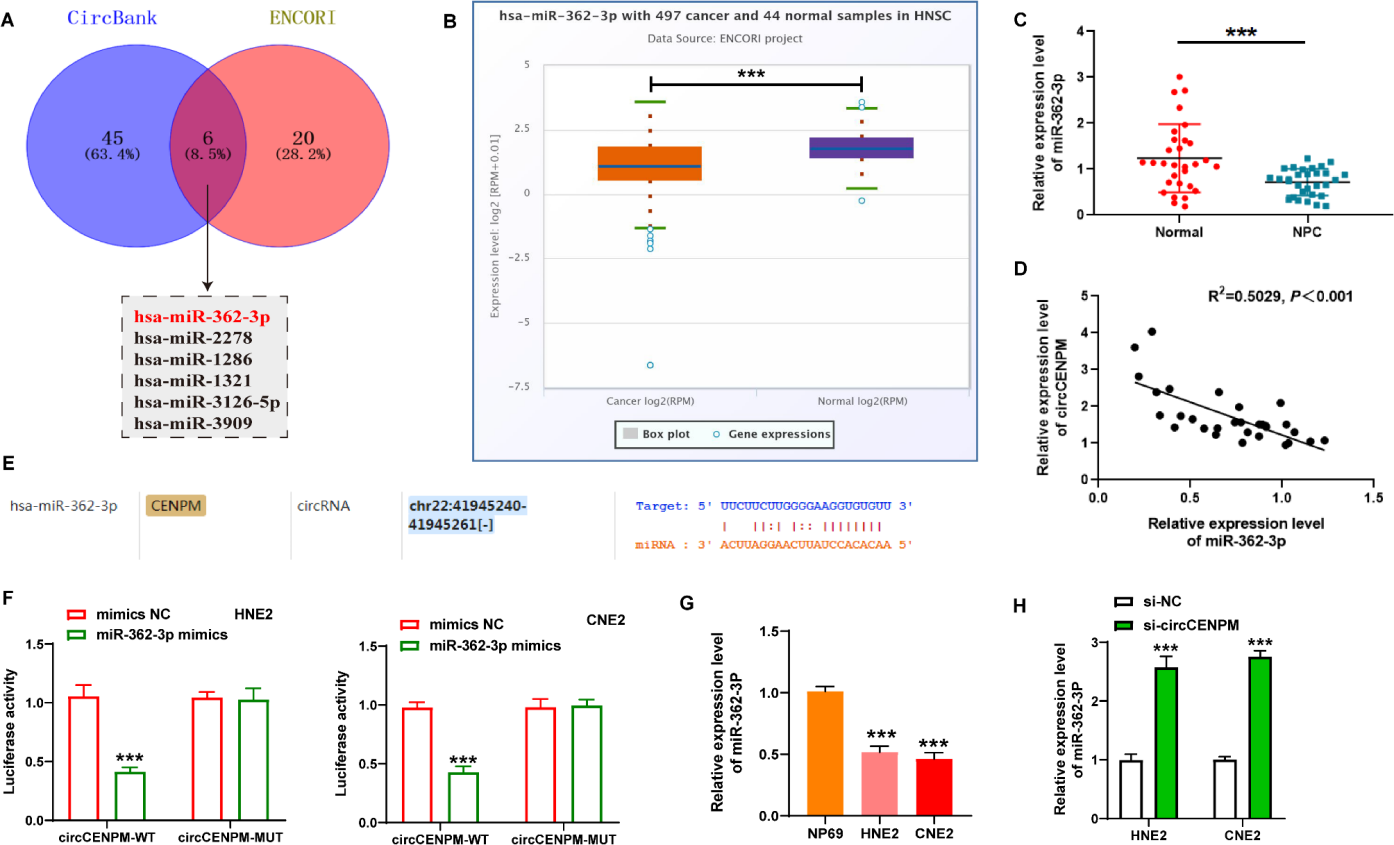
CircCENPM directly bound to miR-362-3p in NPC cells. (**A**) The potential miRNAs of circCENPM were predicted by Circbank and ENCORI online databases. (**B**) The expression of miR-362-3p in Head-and-neck Carcinoma was revealed by ENCORI. (**C**) qRT-PCR disclosed that miR-362-3p was diminished in NPC tissues (*n* = 30). (**D**) The linear relationship between miR-362-3p and circCENPM was assessed using Pearson’s analysis. (**E**) The predicted binding sites between circCENPM and miR-362-3p were analyzed through ENCORI. (**F**) The binding relationship between circCENPM and miR-362-3p was validated using dual-luciferase reporter assay. (**G**) qRT-PCR showed that miR-362-3p was mitigated in NPC cells. (**H**) The miR-362-3p level was monitored after circCENPM silencing. ***P < 0.001

So far, multiple research have disclosed that circRNAs serve as “miRNA sponges” that modulate the expression of downstream miRNAs, thereby inducing various effects on biological behavior of tumor cells [18, 19]. Thus, we predicted the possible circCENPM-associated miRNAs by bioinformatic analysis (Circbank and ENCORI online databases). After taking intersection, six miRNAs were obtained (Fig. 3A). Among them, we found that miR-362-3p is an obviously downregulated gene in Head-and-neck Carcinoma through ENCORI database (Fig. 3B), and previous research have confirmed that abnormally declined miR-362-3p contributes to the screening and diagnosis of NPC [20]. Here, our research also validated the low levels of miR-362-3p in NPC tissues (Fig. 3C), and it was negatively correlated with the expression of circCENPM (Fig. 3D). The analysis result from ENCORI displayed the binding sites between miR-362-3p and circCENPM (Fig. 3E). So, we further validated the targeting relationship between them based on dual-luciferase reporter gene detection. The data exhibited that luciferase activity of circCENPM-WT reporter was diminished by introduction of miR-362-3p mimic, whereas no changes were monitored in circCENPM-MUT reporter (Fig. 3F). Subsequently, we assessed miR-362-3p expression in NPC at cellular levels. qRT-PCR data showed that miR-362-3p was dramatically reduced in HNE2 and CNE2 cells (Fig. 3G). Also, we validated the reverse regulation of the two at the cellular level, miR-362-3p level was elevated after circCENPM silencing (Fig. 3H). Overall, above results disclosed that miR-362-3p was directly targeted by circCENPM and notably downregulated in NPC.

### CircCENPM knockdown inhibited malignant process of NPC cell depending on miR-362-3p

**Fig. 4:**
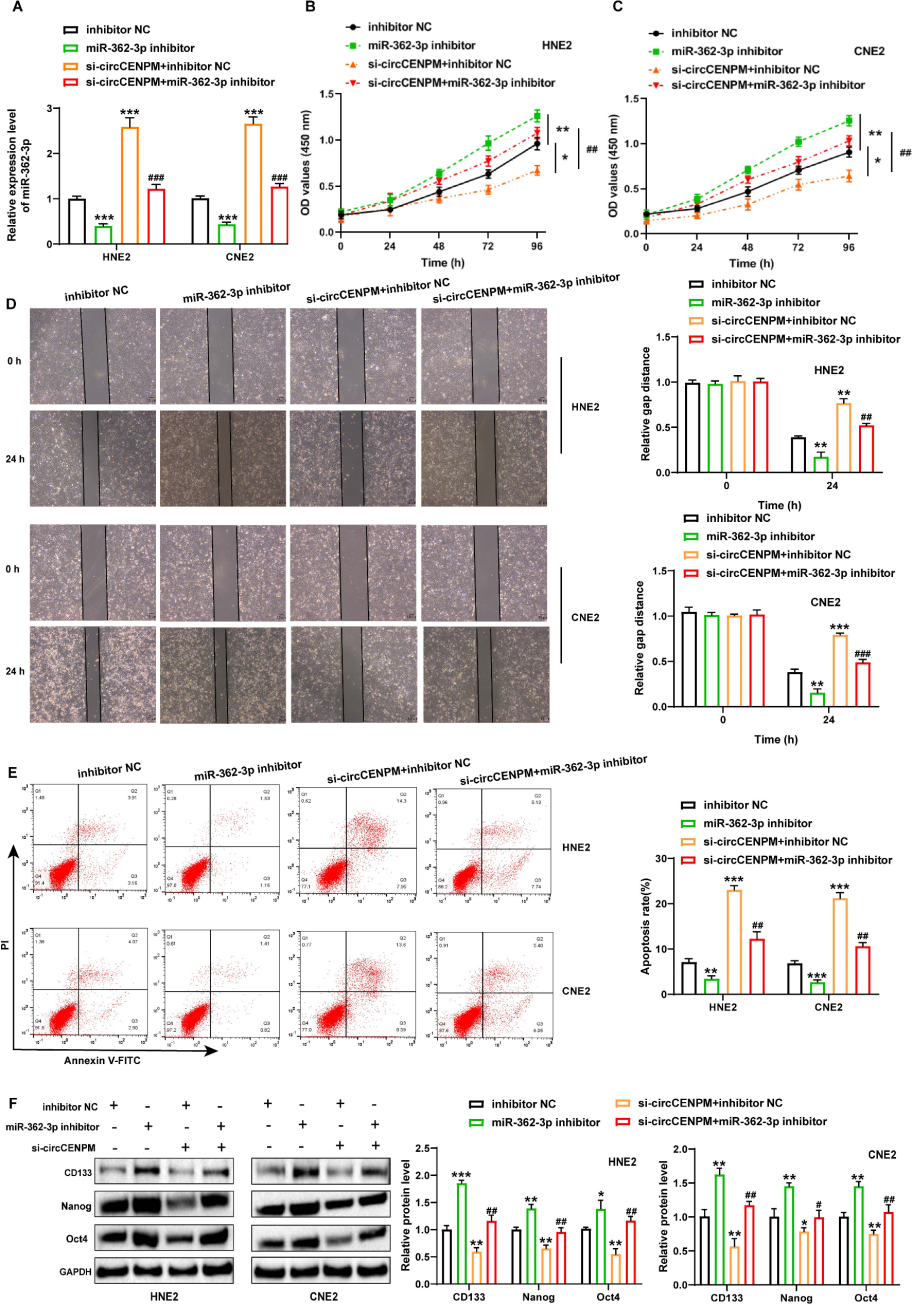
CircCENPM knockdown inhibited malignant process of NPC cell depending on miR-362-3p. NPC cells were transfected with inhibitor-NC, miR-362-3p inhibitor, si-circCENPM + inhibitor-NC or si-circCENPM + miR-362-3p inhibitor. (**A**) miR-362-3p expression was identified by qRT-PCR. (**B-F**) The changes of NPC cell viability, migration, apoptosis and stemness markers were checked through CCK-8 (**B-C**), wound healing (**D**), flow cytometry (**E**) and western blot (**F**) after treatment as indicated. *P < 0.05, **P < 0.01, ***P < 0.001, ^#^*P* < 0.05, ^##^*P* < 0.01, ^###^*P* < 0.001

Previous results demonstrated that circCENPM could inversely regulated miR-362-3p. To validate whether the presence of miR-362-3p restrained the impacts of circCENPM on NPC, we designed the rescue experiments. According to the experimental purpose, the following groups were designed: inhibitor-NC; miR-362-3p inhibitor; si-circCENPM + inhibitor-NC; si-circCENPM + miR-362-3p inhibitor group. qRT-PCR assays presented that miR-362-3p was notably declined after transfection of miR-362-3p inhibitor, while simultaneous silencing of circCENPM substantially enhanced miR-362-3p (Fig. 4A). Afterward, the impacts of circCENPM/miR-362-3p axis on NPC cell growth, metastasis, and stemness were confirmed. CCK-8 and wound healing detection illustrated that miR-362-3p inhibitor introduction enhanced cell proliferation and migration; however, simultaneous knockdown of circCENPM impeded this phenomenon (Fig. 4B-D). Moreover, flow cytometry experiments disclosed that when miR-362-3p and circCENPM were downregulated simultaneously, the decrease of apoptosis capacity induced by miR-362-3p inhibitor was abolished in NPC cells (Fig. 4E). Furthermore, western blot outcomes indicated that stemness-related protein expression was elevated after inhibiting miR-362-3p, whereas further diminished in response to circCENPM silencing (Fig. 4F). Therefore, we concluded that circCENPM knockdown restrained the growth, metastasis, and stemness of NPC cells by targeting miR-362-3p.

### BMI1 served as the target of miR-362-3p in NPC cells

**Fig. 5:**
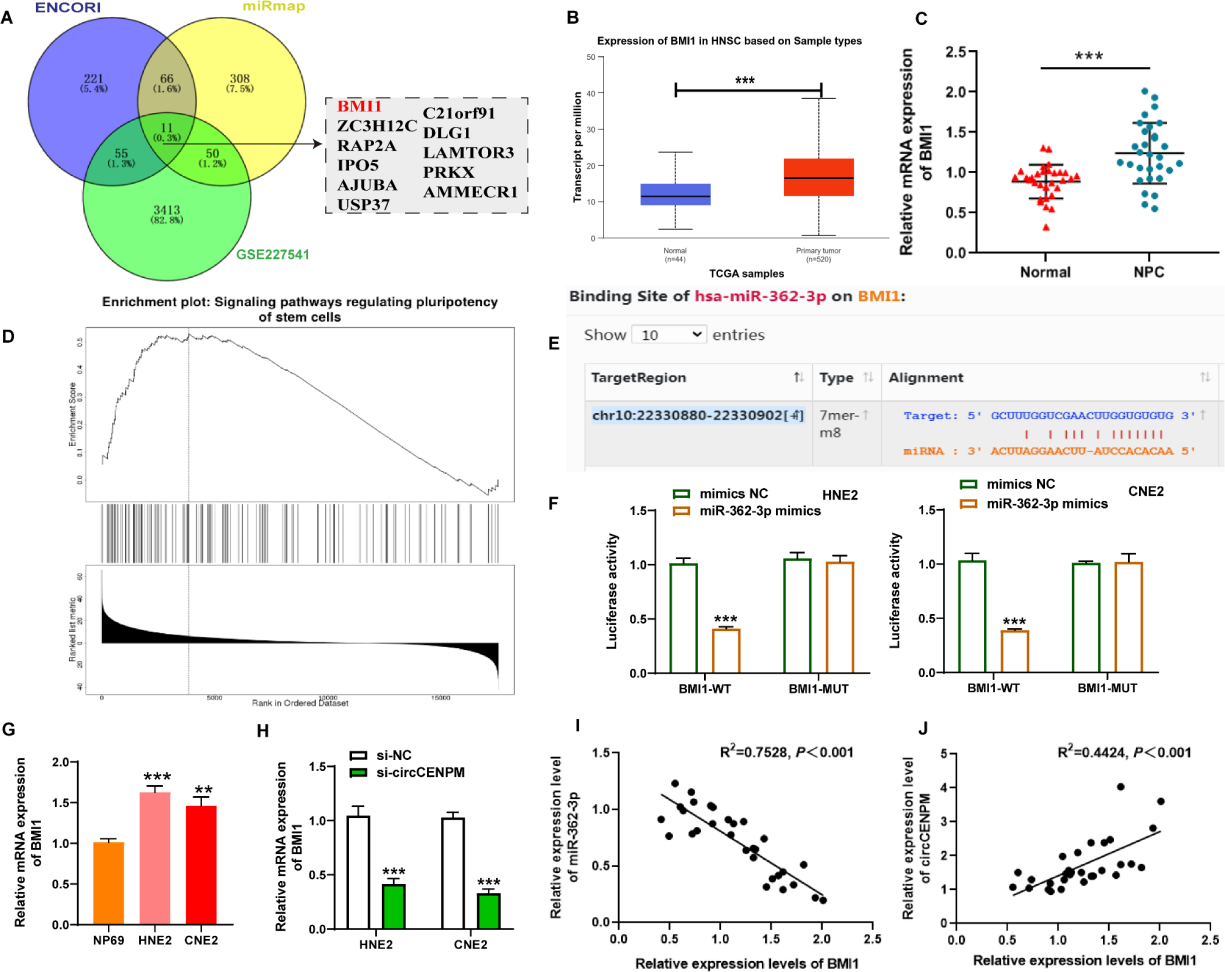
BMI1 served as the target of miR-362-3p in NPC cells. (**A**) The underlying mRNAs were predicted using ENCORI, miRmap and GSE227541 online databases. (**B**) The expression of BMI1 in Head-and-neck Carcinoma was revealed by ENCORI. (**C**) qRT-PCR was executed to validate BMI1 mRNA expression in NPC tissues. (**D**) Molecular function enrichment analysis for BMI1 co-expressed genes in Head-and-neck Carcinoma based on LinkedOmics database. (**E**) The predicted binding sites between miR-362-3p and BMI1 3’UTR were predicted by ENCORI. (**F**) The binding relationship between miR-362-3p and BMI1 were confirmed via dual-luciferase reporter assay. (**G**) qRT-PCR was conducted to identify BMI1 mRNA expression in NPC cells. (**H**) The mRNA level of BMI1 after circCENPM knockdown in NPC cells was measured using qRT-PCR. (**I**) The linear relationship between miR-362-3p and BMI1 mRNA expression in NPC tissues was evaluated through Pearson’s analysis. (**J**) The linear relationship between circCENPM and miR-362-3p expression in NPC tissues was assessed via Pearson’s analysis. **P < 0.01, ***P < 0.001

The above research confirmed that circCENPM could mediate the biological process of NPC cells by regulating miR-362-3p. To further expand the molecular regulatory network of circCENPM/miR-362-3p, we probed the underlying mRNAs using ENCORI, miRmap, and GSE227541 online databases. 11 mRNAs were analyzed from the results in intersection (Fig. 5A). Among them, ENCORI database and tumor samples first suggested the low expression of BMI1 in NPC (Fig. 5B-C). Then, we performed a functional enrichment analysis using LinkedOmics database to reveal the biological function of BMI1. Interestingly, we found that BMI1 is positively involved in regulating stemness-related pathways in Head-and-neck Carcinoma (Fig. 5D), so we predicted that BMI1 may be a key downstream mRNA for circCENPM to augment the growth, metastasis, and stemness of NPC through the ceRNA mechanism. Meanwhile, we found complementary sites of miR-362-3p in the 3’UTR region of BMI1 through ENCORI (Fig. 5E). Then dual-luciferase reporter assay was conducted to validate the interaction between BMI1 and miR-362-3p. The results exhibited that luciferase activity was substantially mitigated in HNE2 and CNE2 cells after cotransfection of BMI1-WT and miR-362-3p mimic (Fig. 5F), disclosing that miR-362-3p and BMI1 had a binding relationship. Subsequently, we evaluated the expression profiles of BMI1 in NPC at cellular levels. qRT-PCR data indicated that BMI1 was also upregulated in NPC cells (Fig. 5G). Moreover, silencing of circCENPM apparently mitigated BMI1 mRNA expression (Fig. 5H). Furthermore, correlation analysis disclosed that BMI1 was inversely related to miR-362-3p, as well as positively linked to circCENPM in NPC tissues (Fig. 5I-J). In summary, BMI1 was a target of miR-362-3p, and positively modulated by circCENPM.

### miR-362-3p modulated malignant phenotype of NPC cells by targeting BMI1

**Fig. 6:**
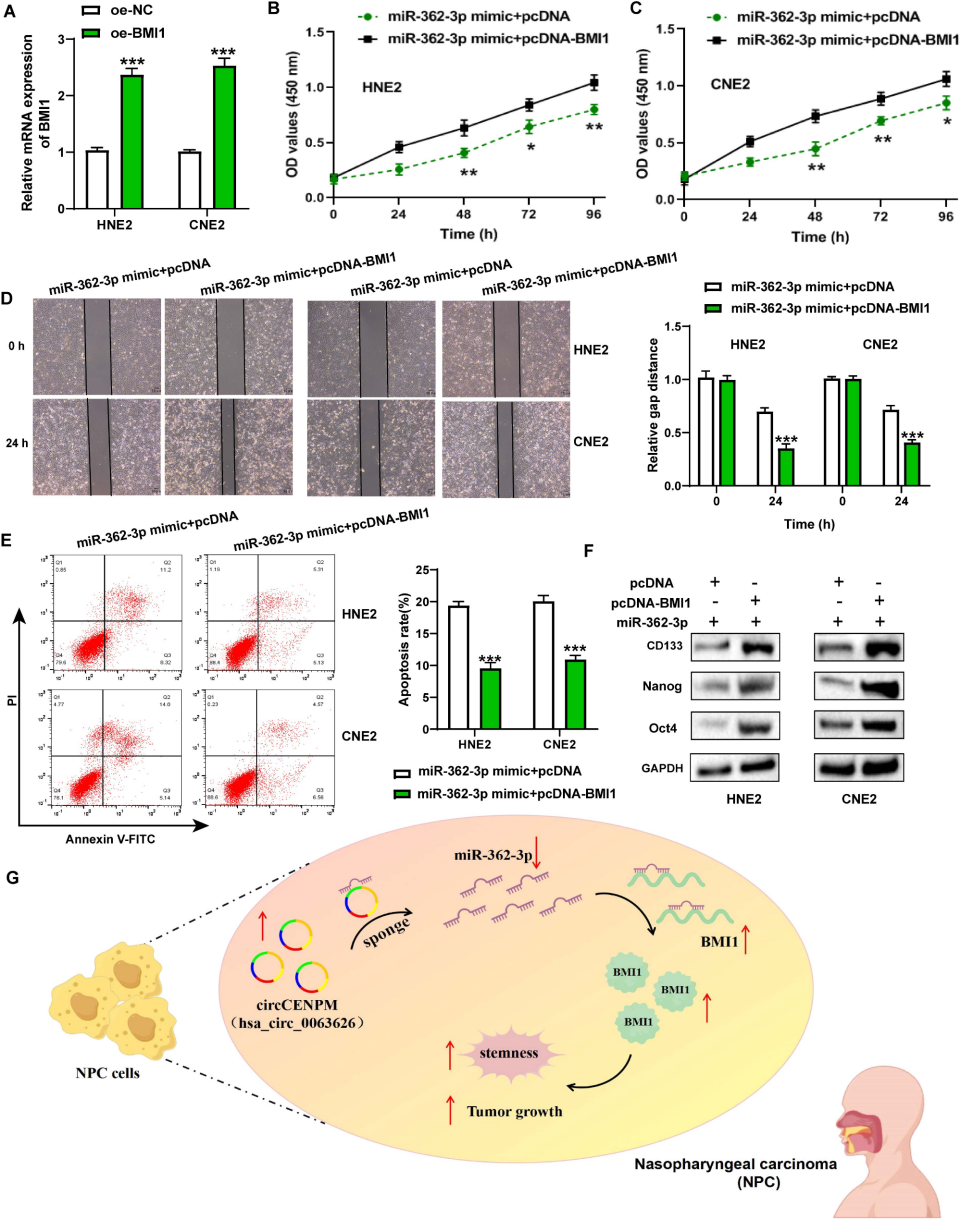
miR-362-3p modulated malignant phenotype of NPC cells by targeting BMI1. (**A**) The BMI1 expression was examined after transfection. (**B-C**) CCK-8, (**D**) wound healing, (**E**) flow cytometry, and (**F**) western blot experiments were employed to measure NPC cell viability, migration, apoptosis, and stemness. (**G**) The schematic diagram presented the mechanism underlying circCENPM as a ceRNA to regulate NPC growth and stemness. *P < 0.05, **P < 0.01, ***P < 0.001

We next executed rescue experiments to validate whether miR-362-3p regulated NPC progression by targeting BMI1. HNE2 and CNE2 cells were transfected with miR-362-3p mimic + pcDNA or miR-362-3p mimic + pcDNA-BMI1. PCR data revealed that BMI1 expression was enhanced in NPC cells after transfection with BMI1 overexpression vector (Fig. 6A). Moreover, functional experiments exposed that NPC cell growth, migration, and stemness properties were facilitated in the miR-362-3p mimic + pcDNA-BMI1 group in contrast to the miR-362-3p mimic + pcDNA group (Fig. 6B-F). These data disclosed that miR-362-3p regulated NPC growth, metastasis and stemness via targeting BMI1.

## Discussion

NPC is a relatively rare malignant tumor. The treatment benefits of NPC patients are still not desirable due to its radiation resistance, strong aggressiveness, and high recurrence rate [6, 21]. In the present work, we identified a novel positive regulator and its mechanism in NPC progression (Fig. 6G), which may put forward novel insights for the NPC diagnosis and treatment of NPC.

CircRNA is a type of closed-loop non-coding RNA with high stability and conservation [7]. Recently, as high-throughput sequencing and bioinformatics technology continues to evolve, emerging circRNAs have been identified to engage in regulating various biological functions in the body. Meanwhile, genome-wide association studies of tumor tissues have disclosed massive circRNAs related to malignant tumors [22]. CircRNA is richly expressed in body fluids and exhibits stage specificity during tissue development, making it a candidate star molecule for cancer diagnosis markers [23]. Additionally, extensive reports have revealed that the imbalance of circRNA expression is inextricably linked to biological processes including apoptosis, proliferation, and metastasis of tumor cells, serving a regulatory role in promoting or hindering tumor progression [12, 24]. Wu et al. identified that circ_0008234 was substantially enhanced in colon cancer, its upregulation facilitated tumor growth and aggressiveness [25]. Luo et al. reported that circ-OXCT1 knockdown restrained lung cancer progression through miR-516b-5p/SLC1A5 [26]. Similarly, circRNAs also perform essential roles in the genesis and advancement of NPC [27]. Circ_0000285 has been identified to drive NPC progression via sponging miR-1278 [28]. A recent study validated that circRILPL1 aggravated NPC progression via activating Hippo-YAP pathway [29]. Here, a novel oncogenic circRNA, circCENPM, in NPC was identified. We first demonstrated the potential value of circCENPM in NPC at the clinical level. The results of pathological characteristics disclosed that elevated circCENPM expression was associated with clinical stage and lymphatic metastasis in NPC patients. Also, NPC patients with high circCENPM level exhibited poor survival. These clinical indicators revealed that circCENPM played a carcinogenic role in NPC and was expected to serve as a potential diagnostic and prognostic biomarker. Nevertheless, more NPC samples are still needed for testing in the future. Besides, we also confirmed that circCENPM contributed to the tumorigenesis and stemness of NPC at the cellular level. Thus, our study indicated that targeting circCENPM might be a promising strategy for treating NPC.

Current research indicates that circRNAs in the cytoplasm serves as miRNA sponges is the main approaches to achieve biological functions [18, 19]. Since circRNA molecules are enriched with miRNA response elements (MREs), they can competitively bind to miRNAs and regulate the expression of target genes, thereby modulating biological functions including growth, apoptosis, and metabolism of tumor cells through exerting competitive endogenous RNA (ceRNA) mechanisms [30]. For example, Circ_0000285 contributed to NPC growth and invasion via miR-1278/FNDC3B network [28]. CircCRIM1 facilitated malignant process of NPC cells by sponging miR-422a and upregulate FOXQ1 [31]. Here, we further explored the potential circCENPM-miRNA regulatory network in NPC. Through online databases analysis, we found that circCENPM could interact with miR-362-3p. Notably, miR-362-3p is a crucial tumor-related miRNA, which always exerts tumor suppressive impacts, including ovarian cancer [32], renal cancer [33], lung cancer [34] and etc. In NPC-related reports, miR-362-3p was indicated as an independent prognostic indicator, and engaged to suppress NPC cell metastasis [20]. In this study, we also identified that miR-362-3p was remarkably diminished in NPC and serves as a tumor suppressor. Meanwhile, introduction of miR-362-3p inhibitor could abolish the restrained effect induced by circCENPM silencing on malignant process of NPC cells.

To further probe the molecular regulatory network of circCENPM/miR-362-3p in NPC, we performed bioinformatic analysis to predict BMI1 as an underlying downstream target of miR-362-3p. BMI1 is considered to be a key factor in regulating tumor metastasis and recurrence [35], and its abnormal upregulation can effectively aggravate the epithelial-mesenchymal transition [36], resistance formation [37], and thrombi formation [38]. Additionally, BMI1 also functions as a stem cell factor, which enhances the stem cell phenotype of cancer cells in various malignancies. Notably, BMI1 presented high levels in NPC, while knockdown of BMI1 could decrease NPC cell stemness [39] and elevated radiosensitivity [40]. Consistent with these past reports, our study further validated BMI1 high expression pattern in NPC. It was evidenced that BMI1 introduction crippled miR-362-3p-mediated inhibition on NPC cell growth, metastasis and stemness. Additionally, BMI1 was inversely targeted by miR-362-3p, and positively modulated by circCENPM. Taken together, these findings disclosed the ceRNA regulatory mechanism of circCENPM/miR-362-3p/BMI1 in NPC. However, there are some shortcomings in this study that should be acknowledged. First, more experiments needed to further elucidate the details of NPC cell stemness regulation in future. Second, whether circCENPM can interact RNA-binding proteins to engage NPC progression is also one of our future research directions.

## Conclusion

In summary, our work innovatively validated the carcinogenic role of circCENPM in NPC by facilitating NPC cell growth, metastasis and stemness. Mechanistic results indicated that circCENPM regulated NPC progression via targeting miR-362-3p/BMI1 network. These findings disclosed the circCENPM-mediated bio-functions and molecular mechanism in NPC advancement, providing significant theoretical supports for NPC diagnosis and therapy.

## Electronic supplementary material

Below is the link to the electronic supplementary material.

Supplementary Material 1

Supplementary Material 2

Supplementary Material 3

## Data Availability

The data that support the findings of this study are available from the corresponding author upon reasonable request.

## Abbreviations

NPC: Nasopharyngeal carcinoma
EB: Epstein-Barr
nc RNAs: Non-coding RNAs
miRNA: microRNA
WT: Wild-type
MUT: Mutant-type
