# Supplementary material for: CircCENPM serves as a CeRNA to aggravate nasopharyngeal carcinoma metastasis and stemness via enhancing BMI1
Source: Hereditas. 2025 Mar 14;162:39. doi: 10.1186/s41065-025-00406-7 (PMC11907939; doi:10.1186/s41065-025-00406-7)
Supplement: Supplementary file 1 — Supplementary Material 1 [file 41065_2025_406_MOESM1_ESM.doc]

**Western blot raw images**

**Fig.2E HNE2 cell**


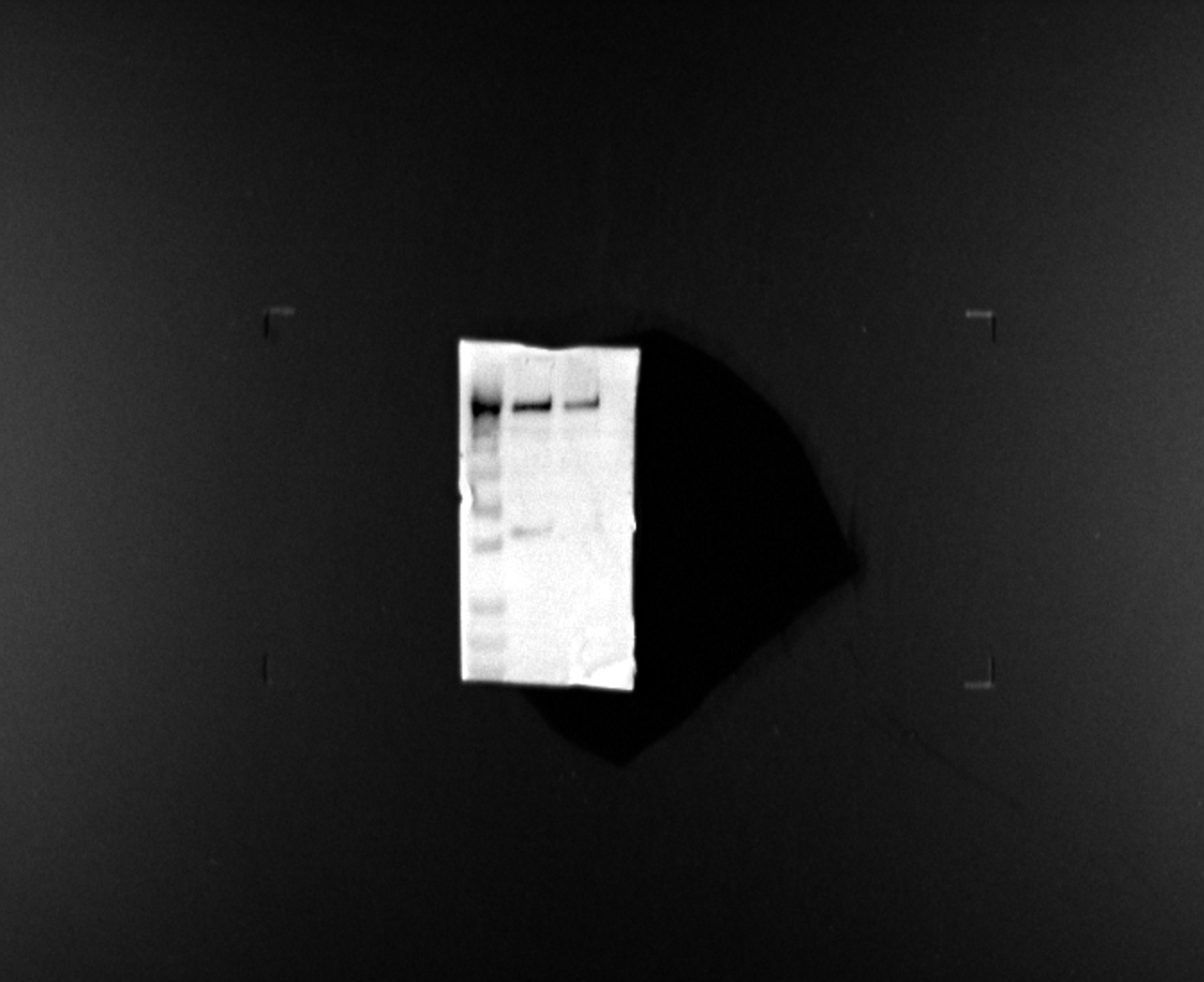


CD133 110 kDa


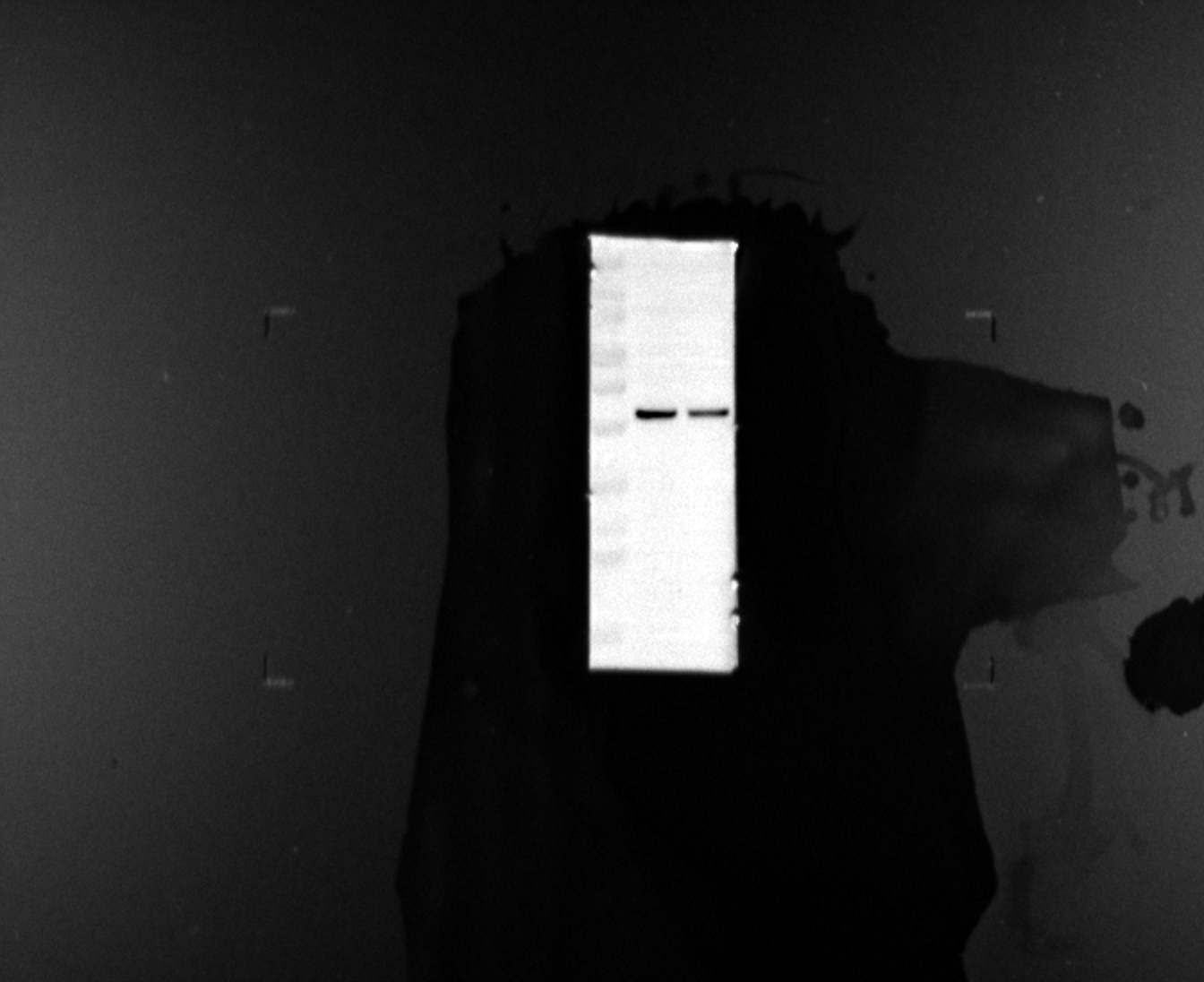


Nanog 42kDa


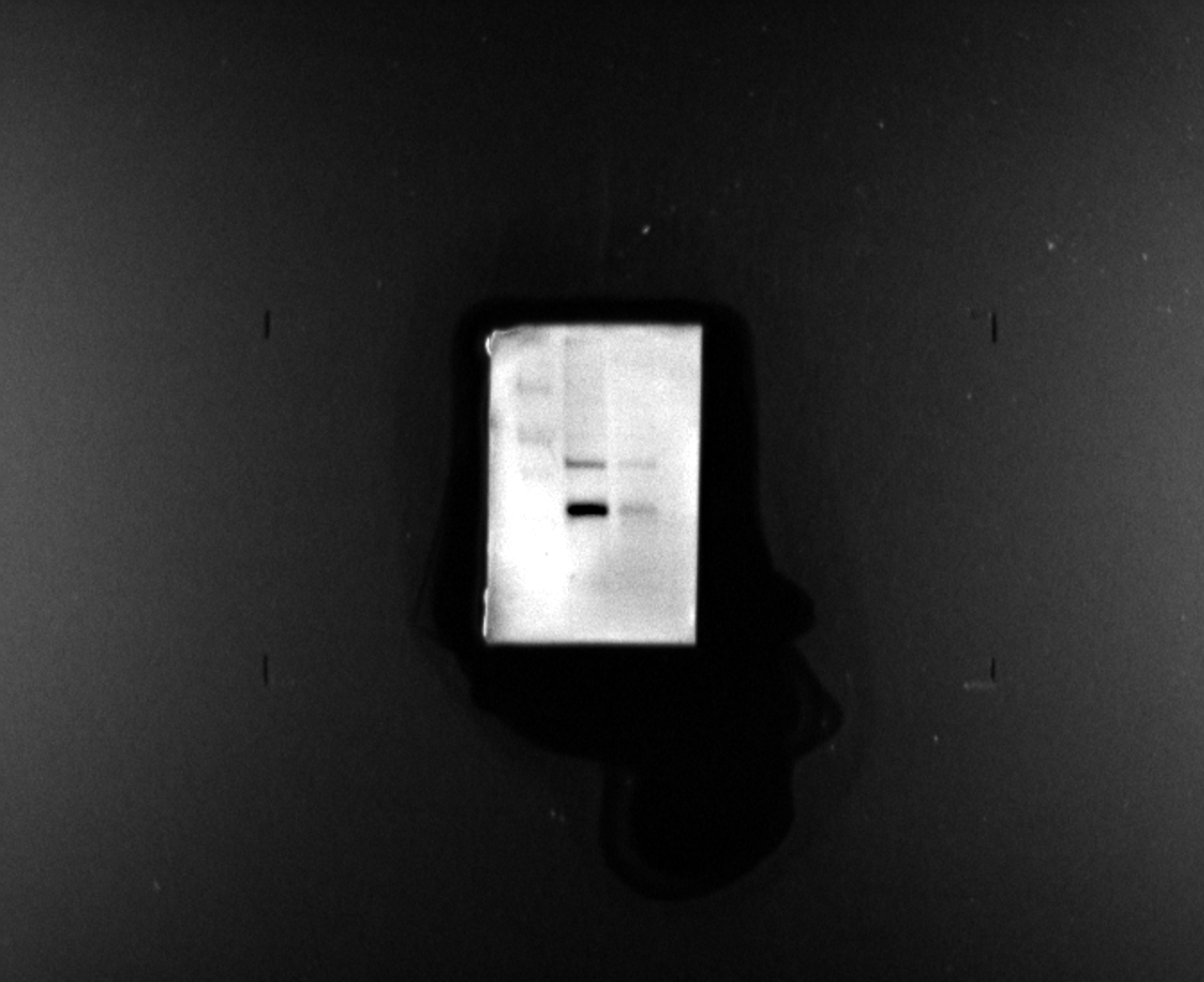


Oct4 45kDa


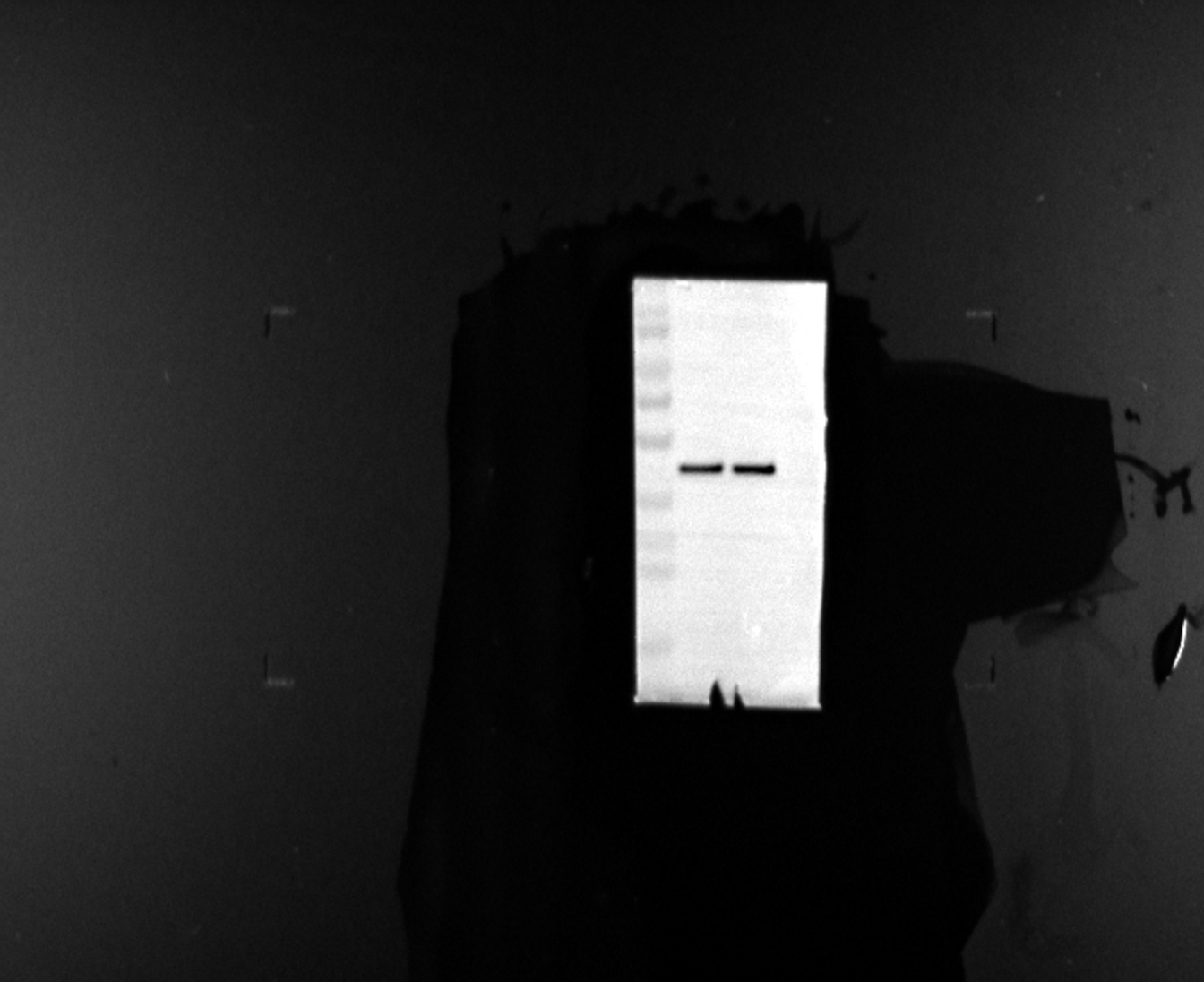


GAPDH 36kDa

**Fig.2E CNE2 cell**


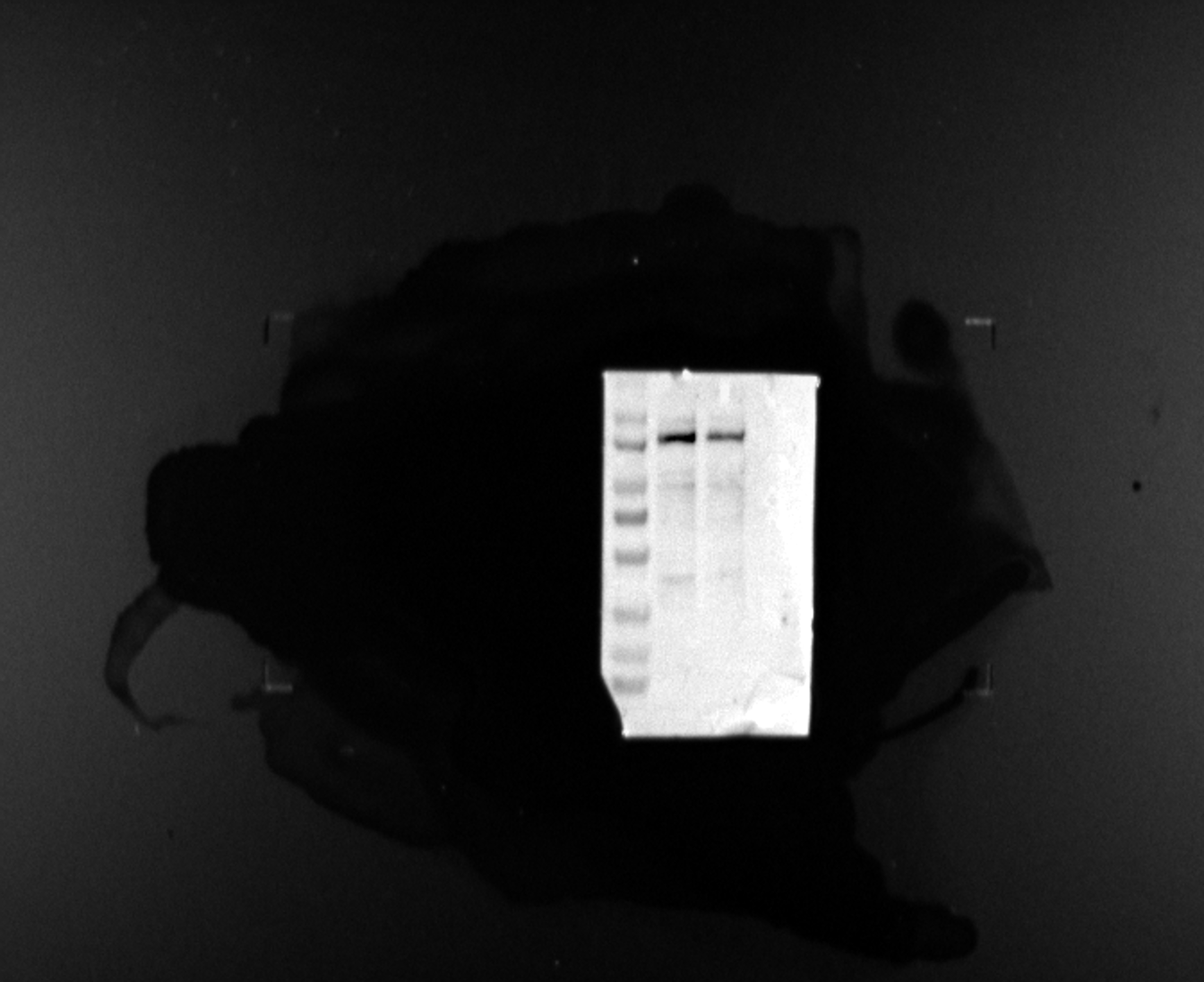


CD133 110 kDa


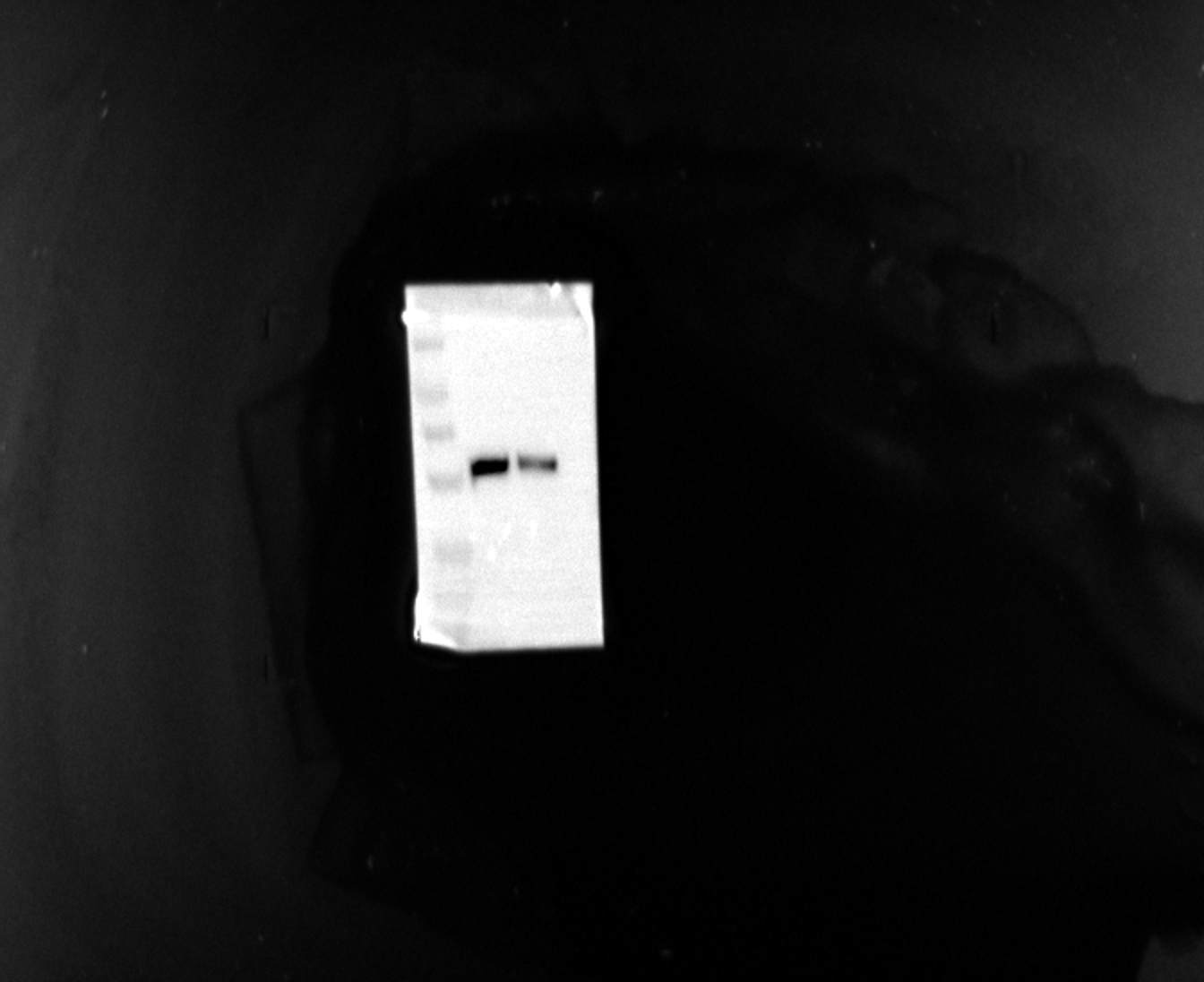


Nanog 42kDa


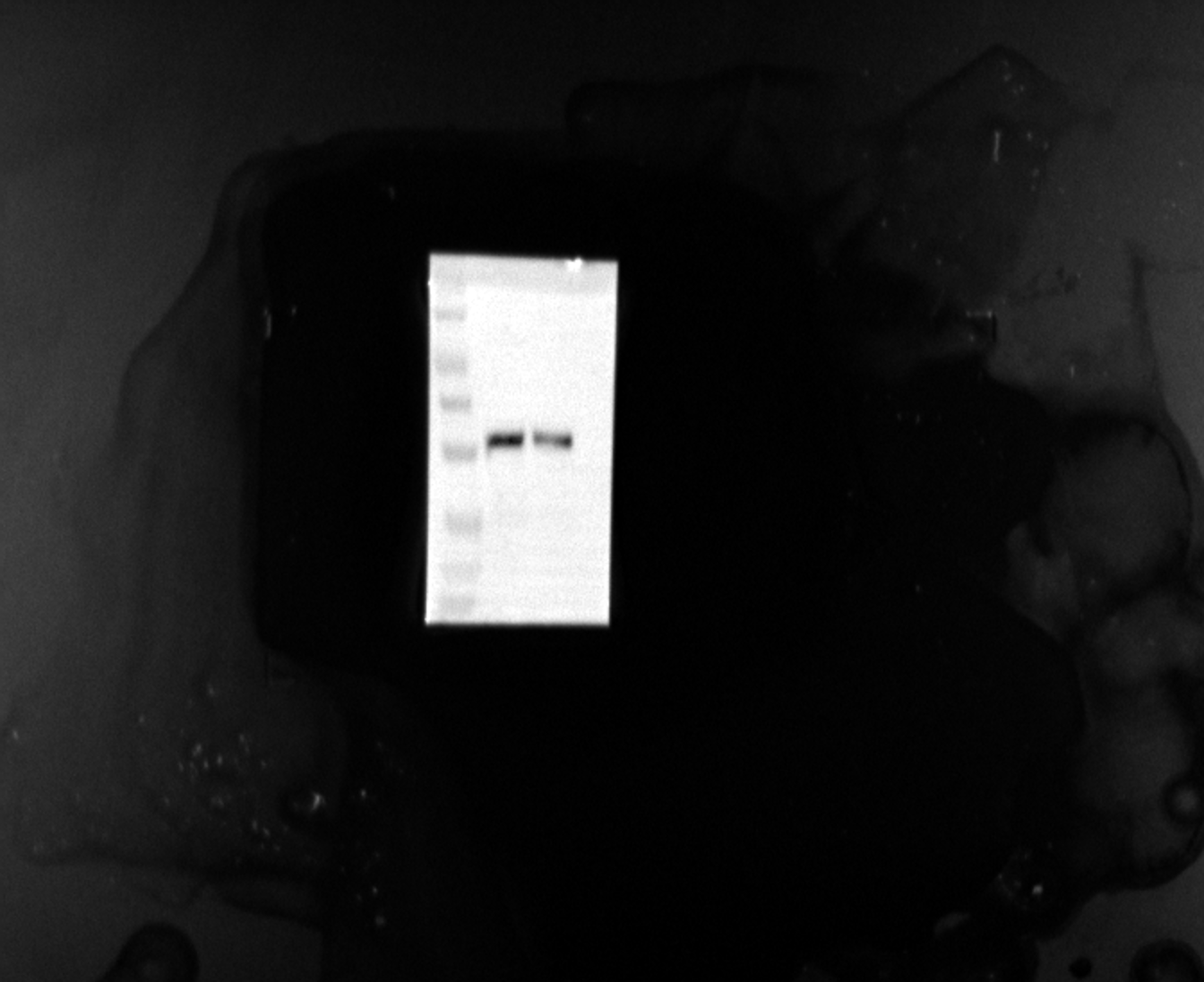


Oct4 45kDa


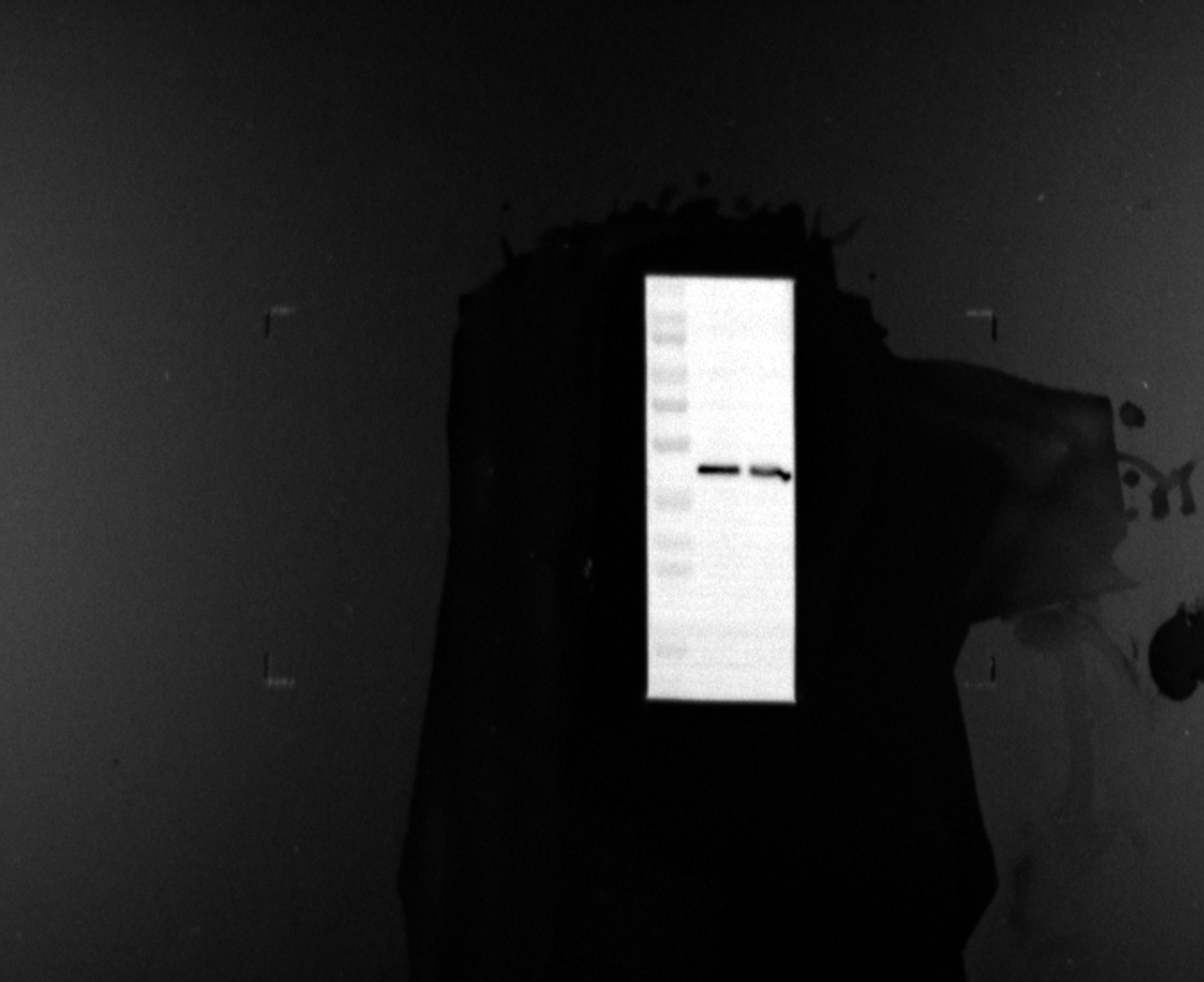


GAPDH 36kDa

**Fig.4F HNE2 cell**

**
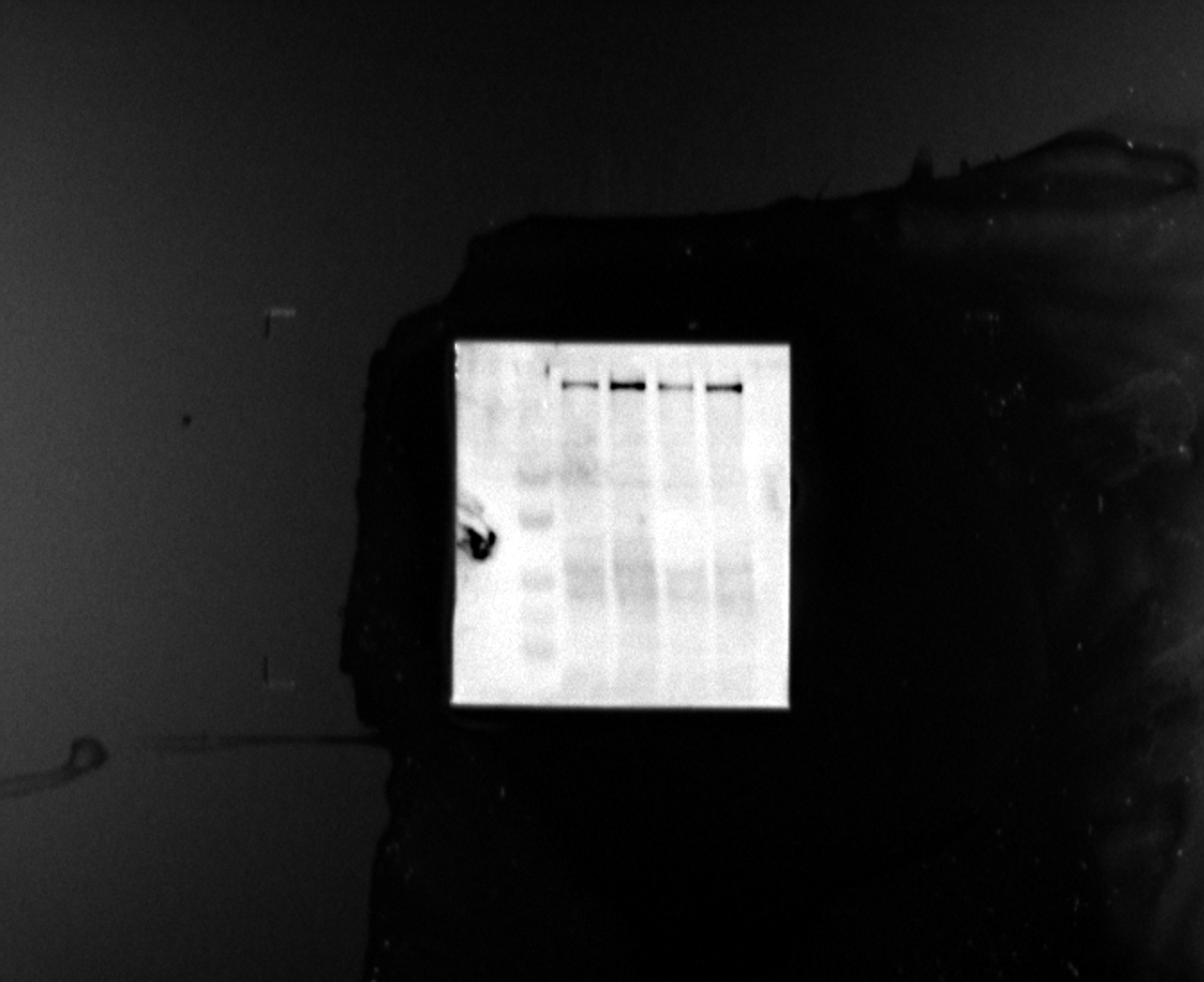
**

CD133 110 kDa

**
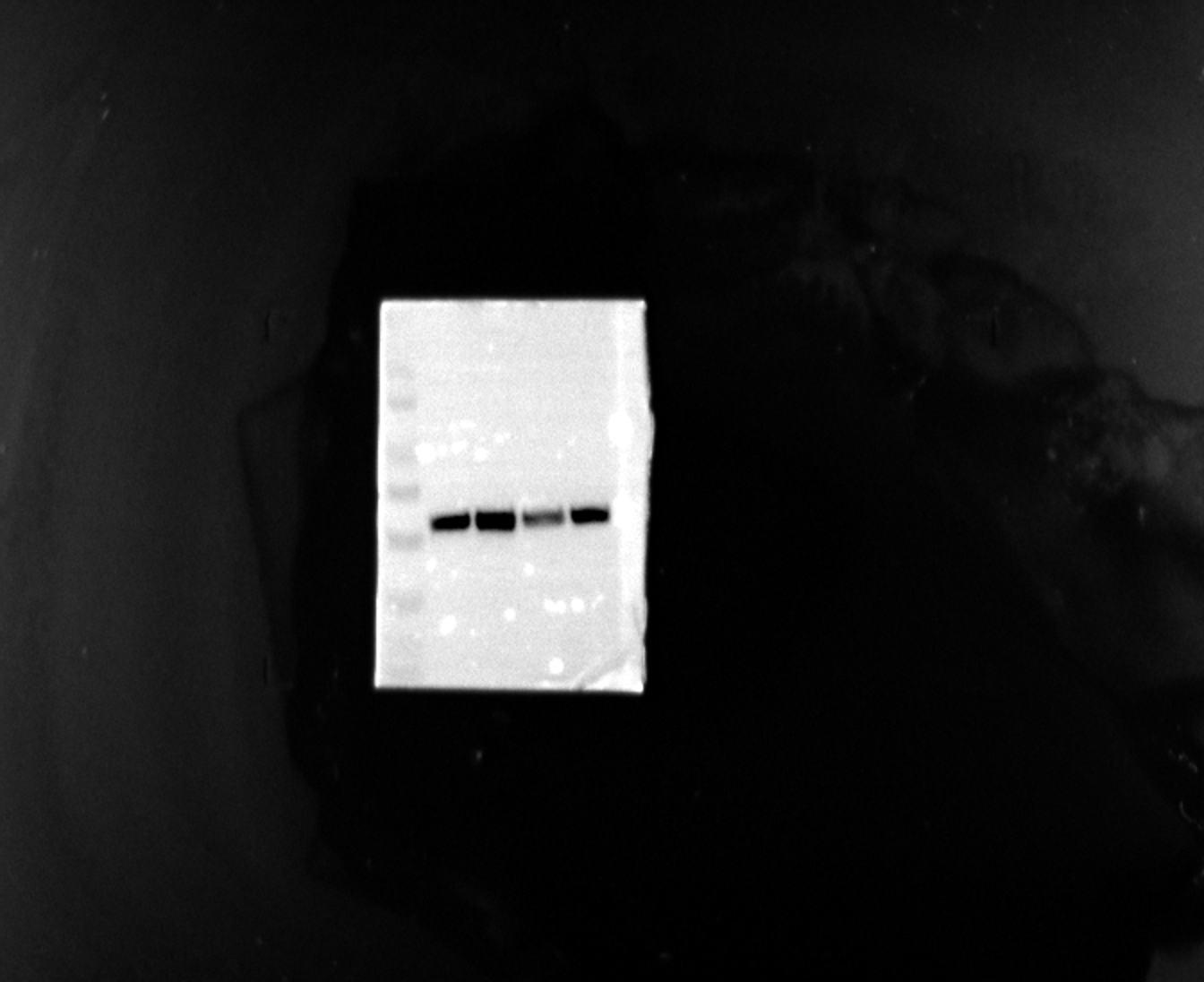
**

Nanog 42kDa

**
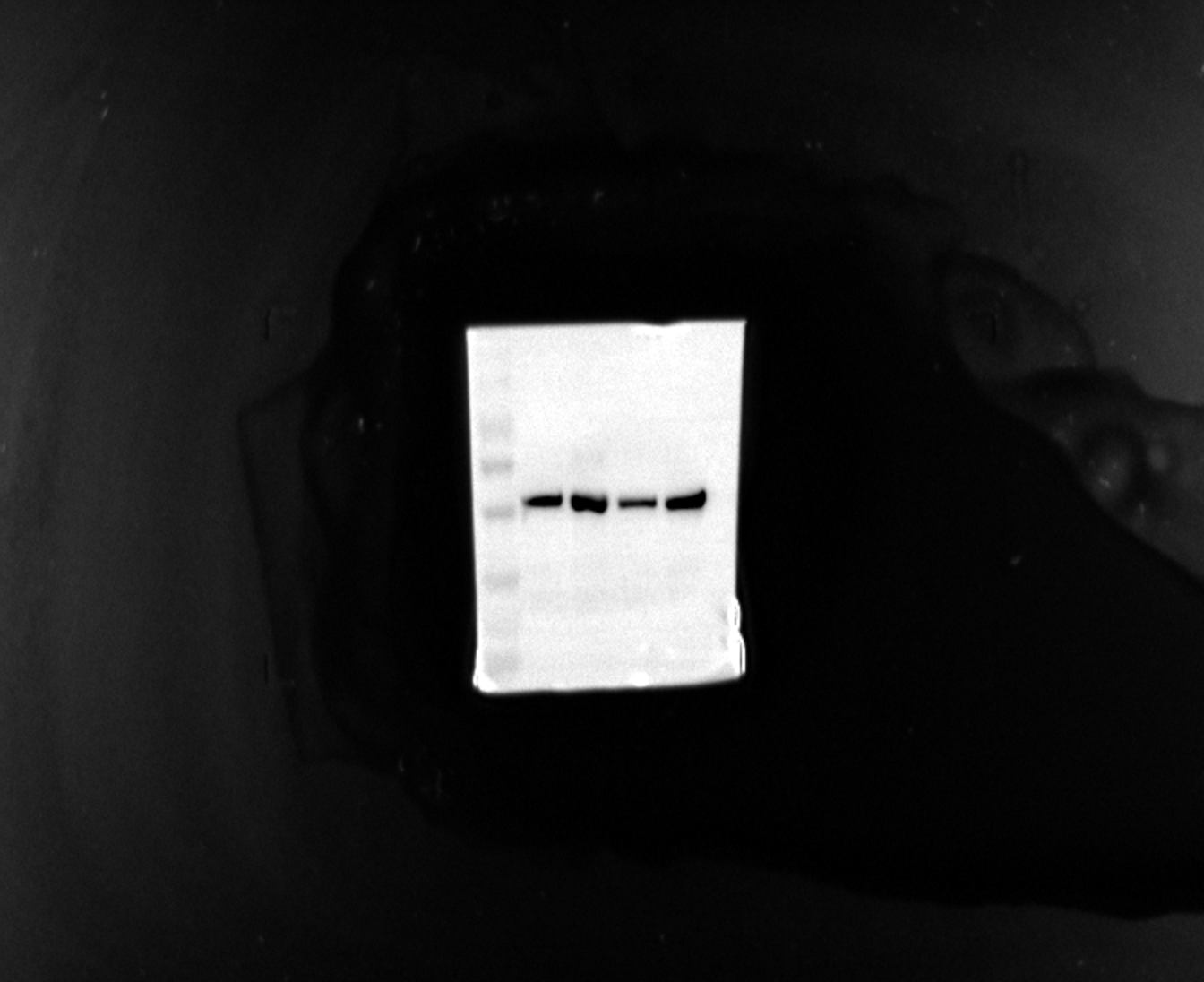
**

Oct4 45kDa

**
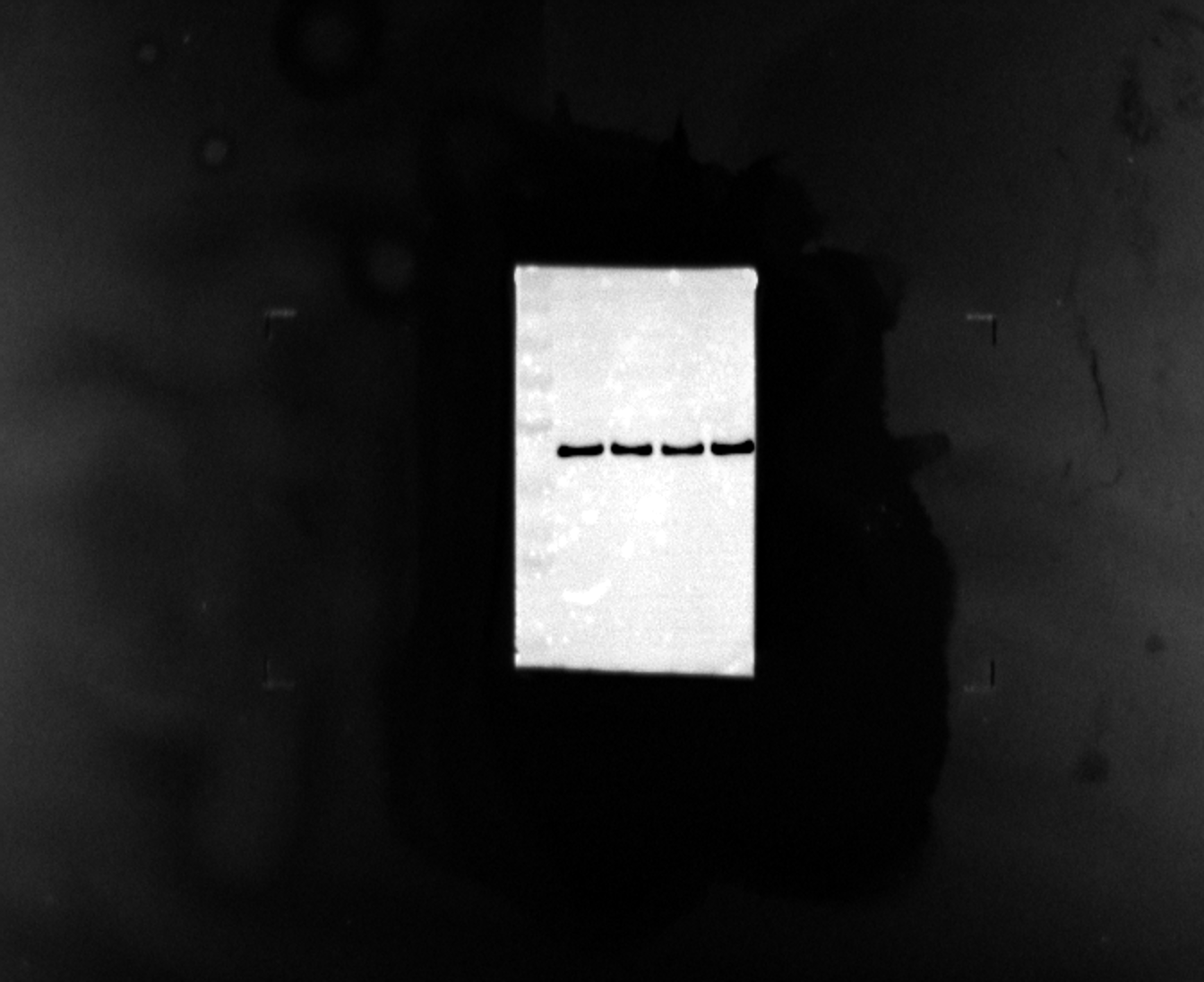
**

GAPDH 36kDa

**Fig.4F CNE2 cell**


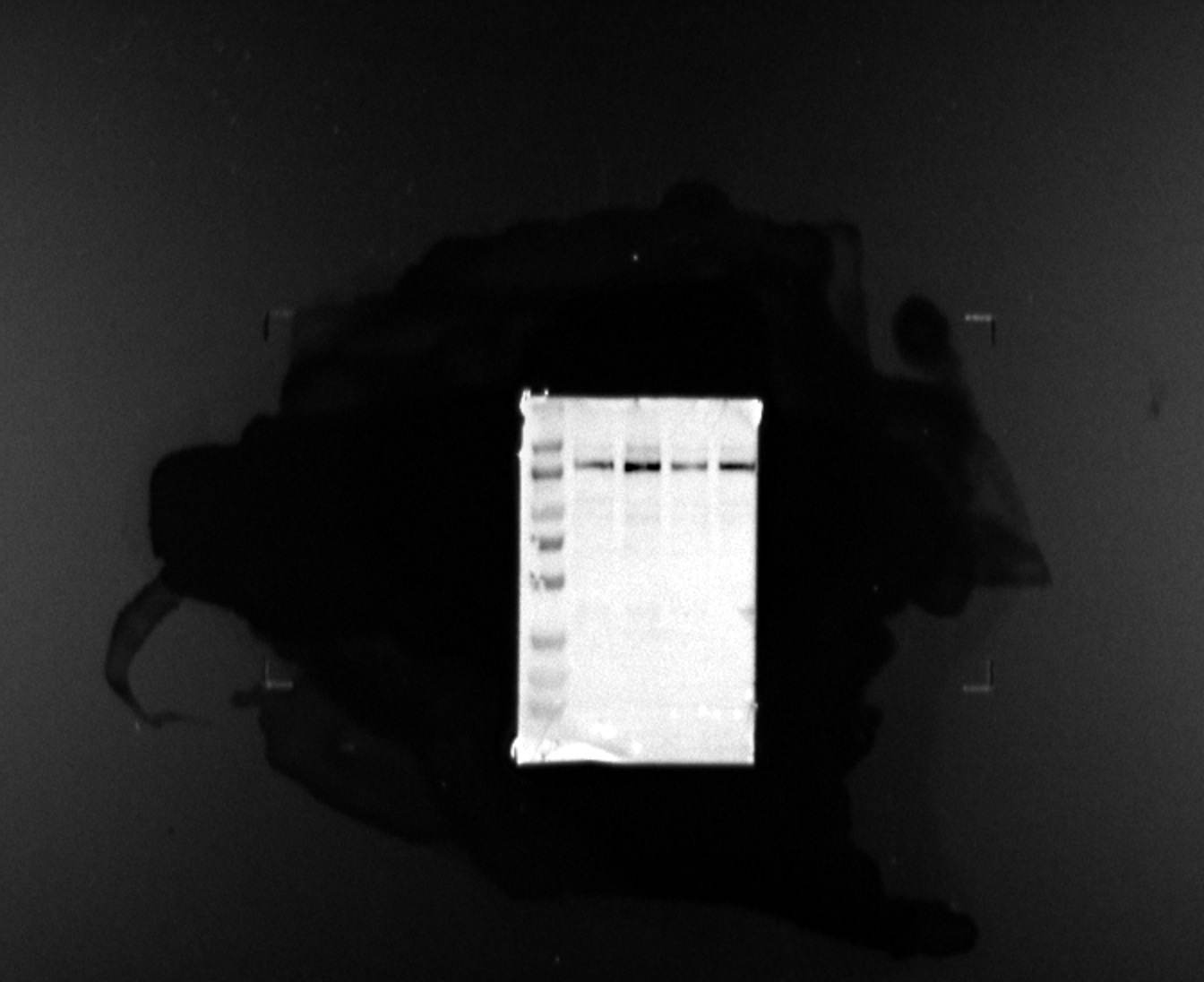


CD133 110 kDa


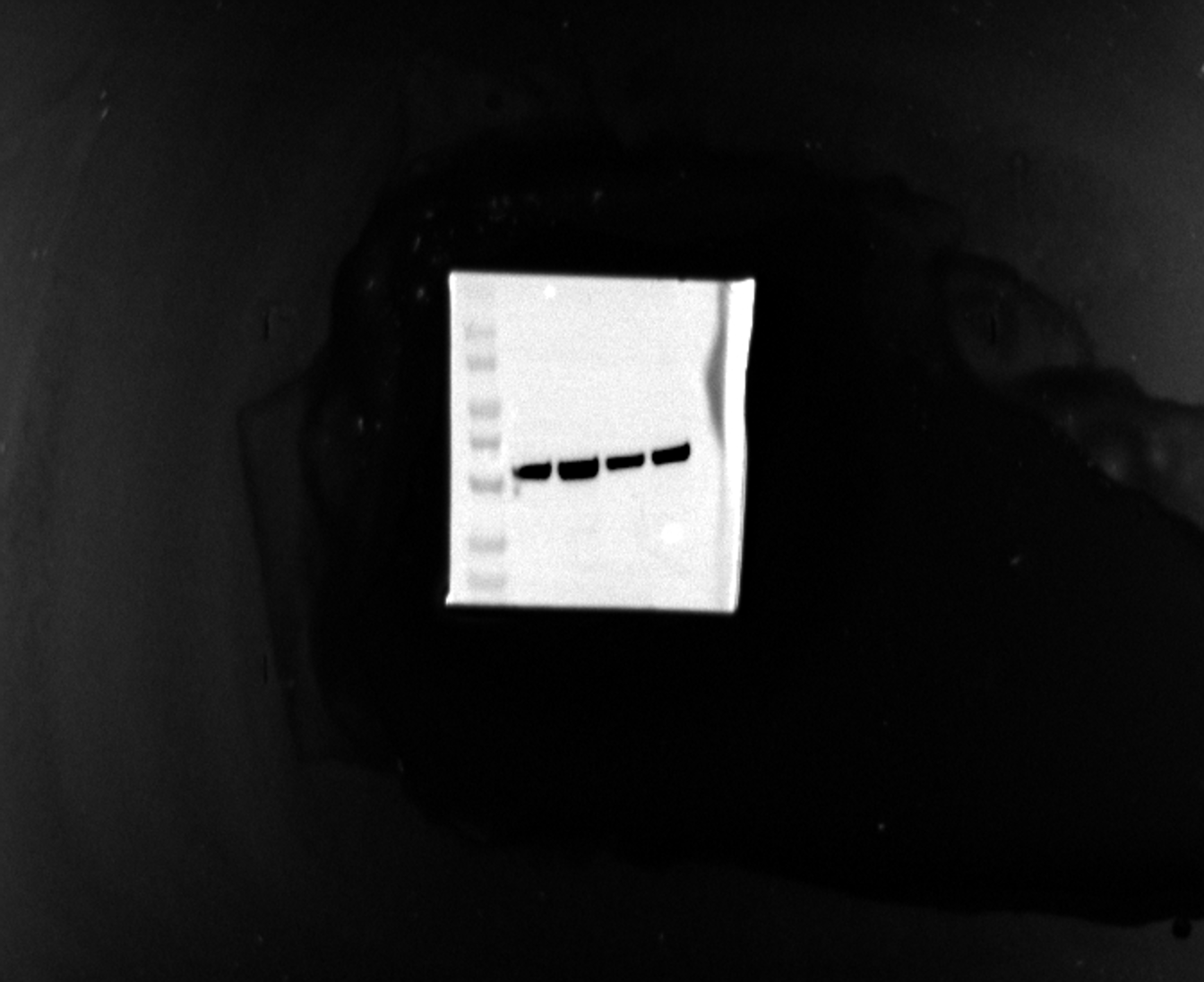


Nanog 42kDa


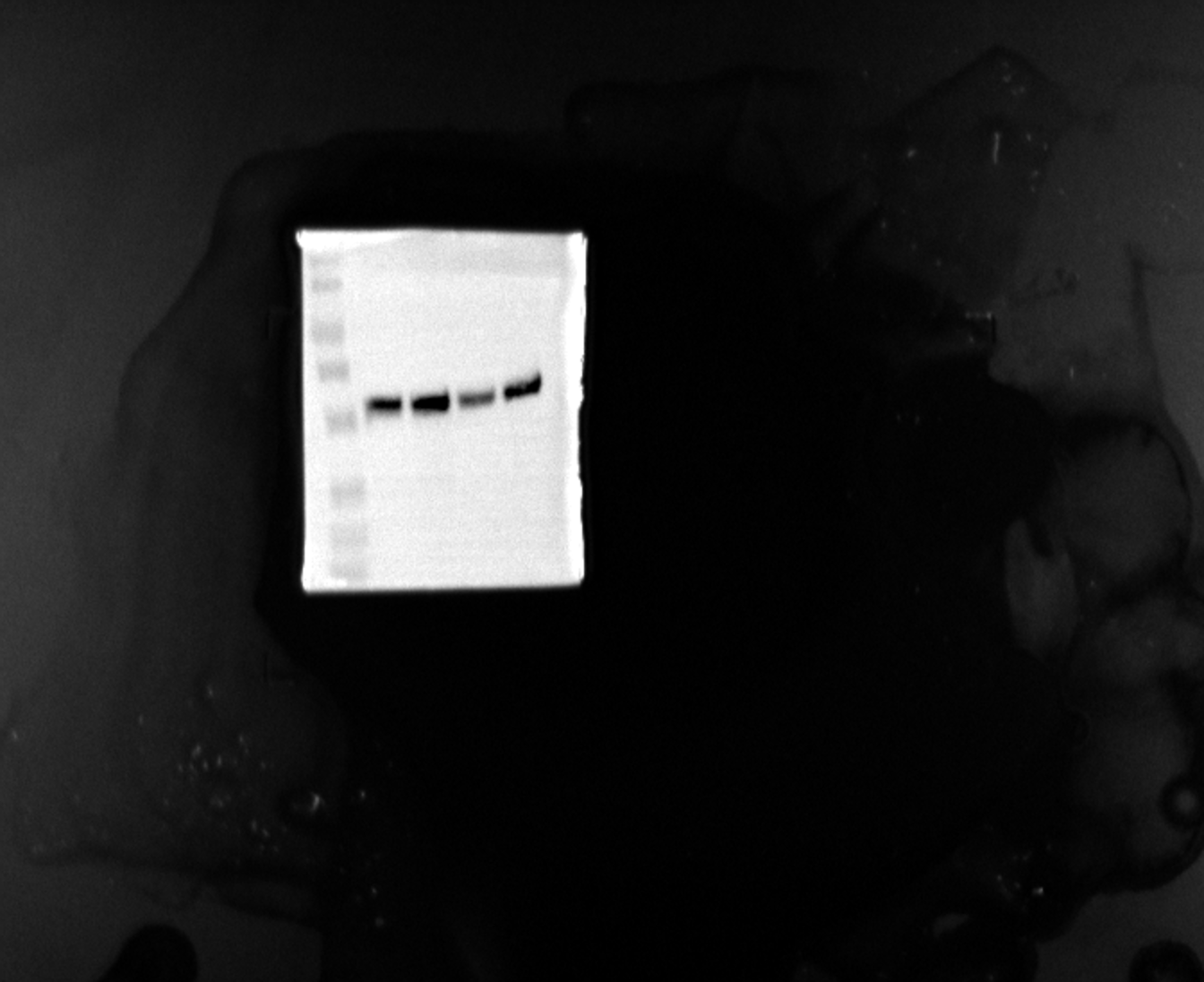


Oct4 45kDa


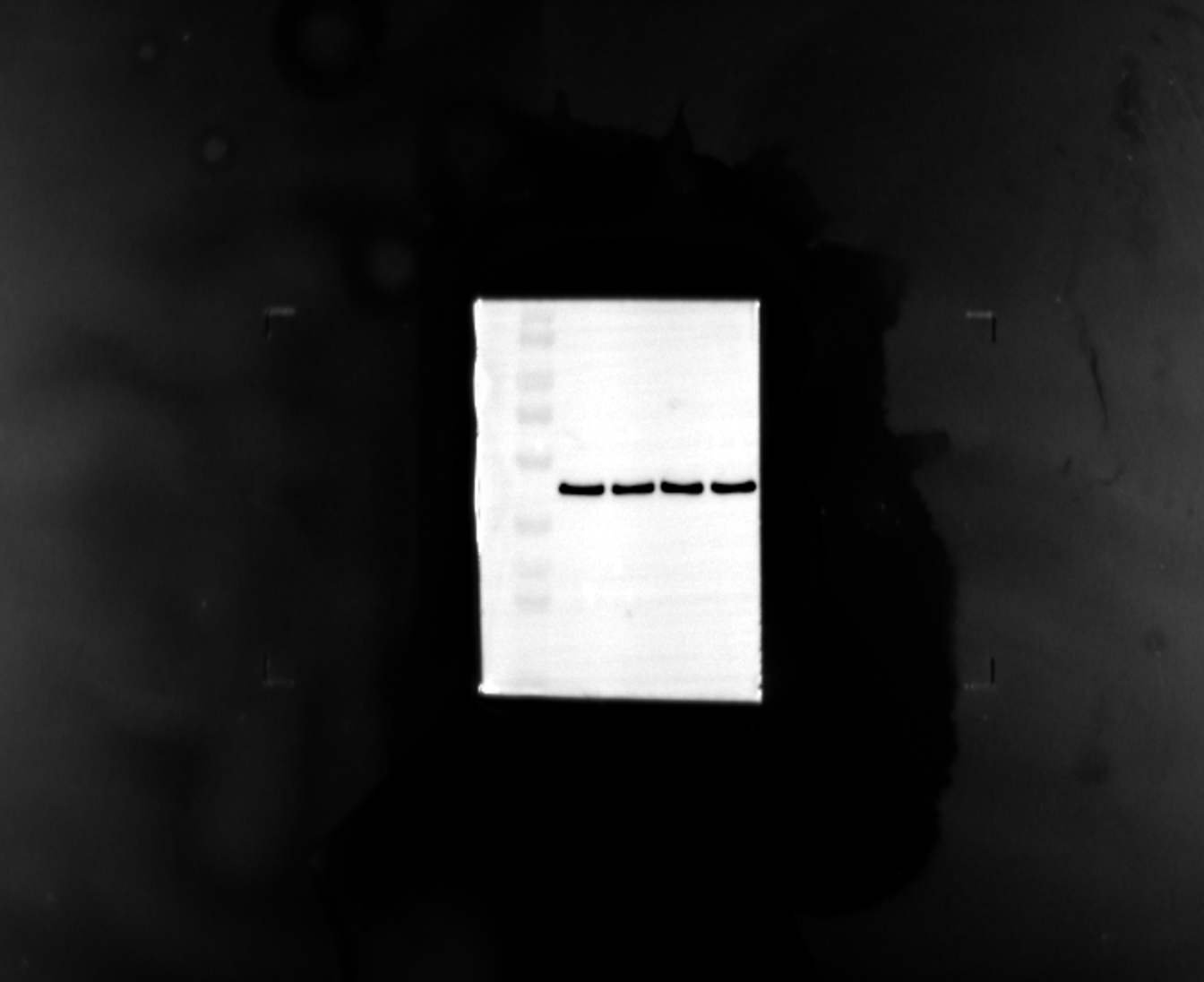


GAPDH 36kDa

**Fig.6F HNE2 cell**

**
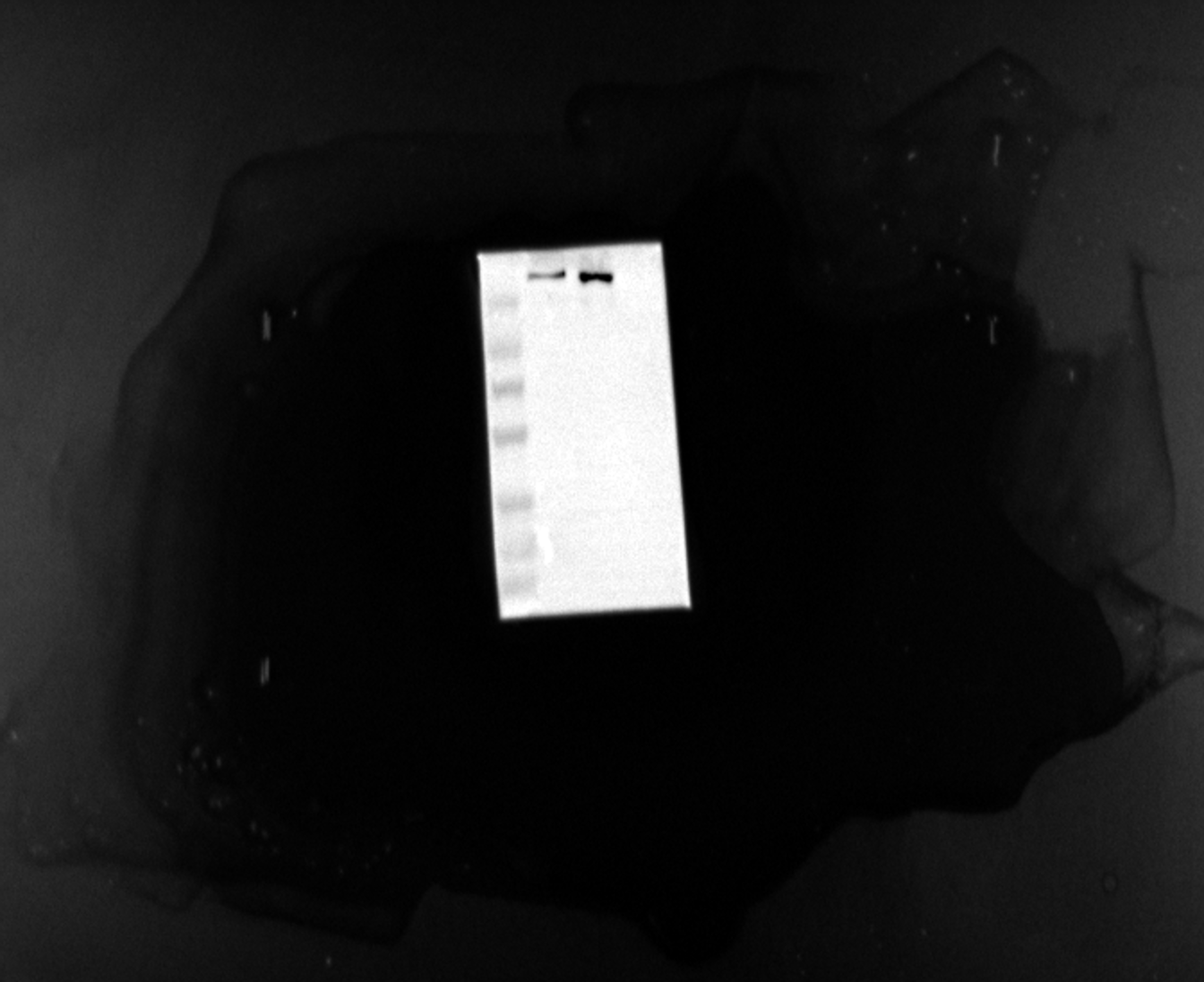
**

CD133 110 kDa

**
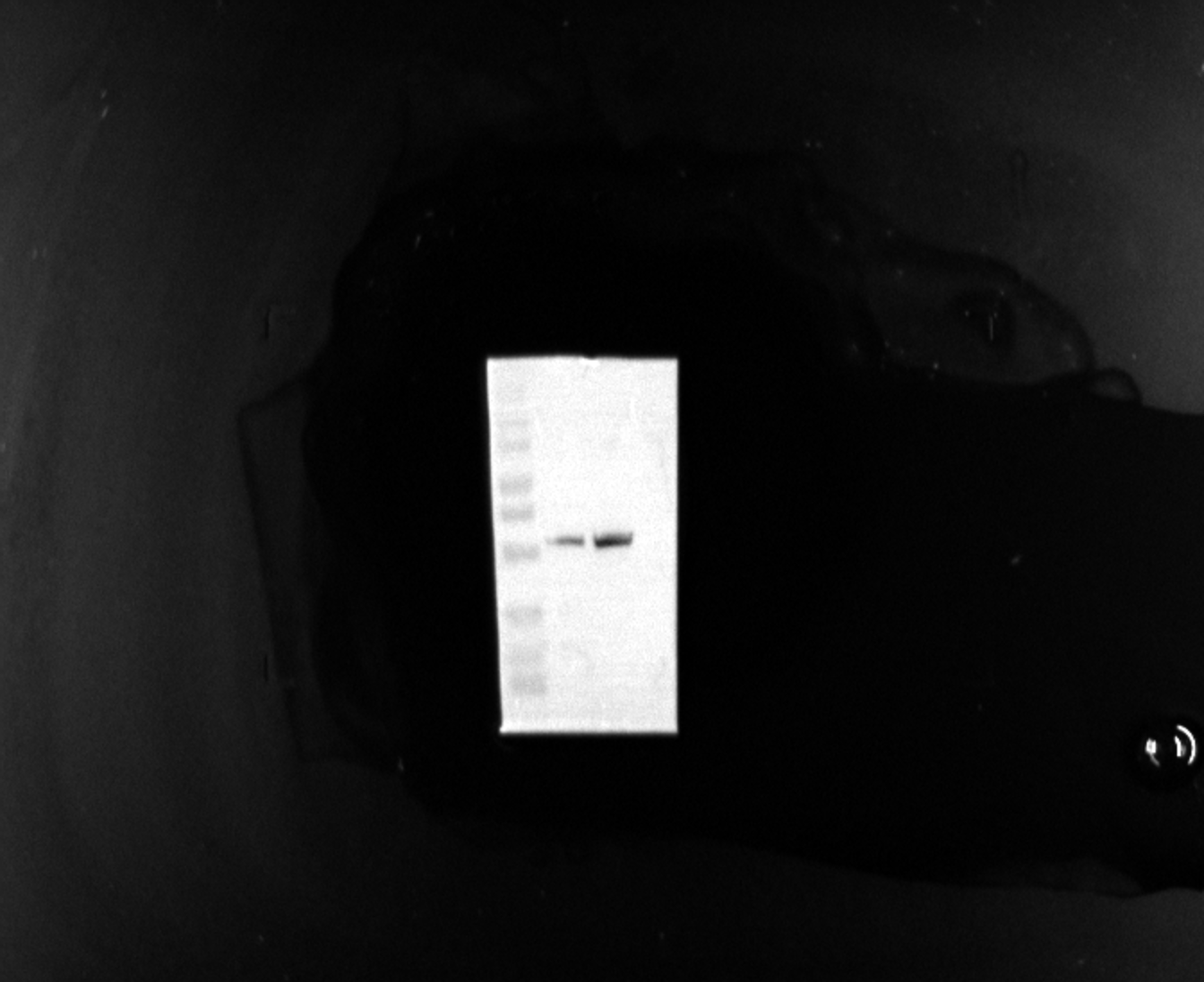
**

Nanog 42kDa

**
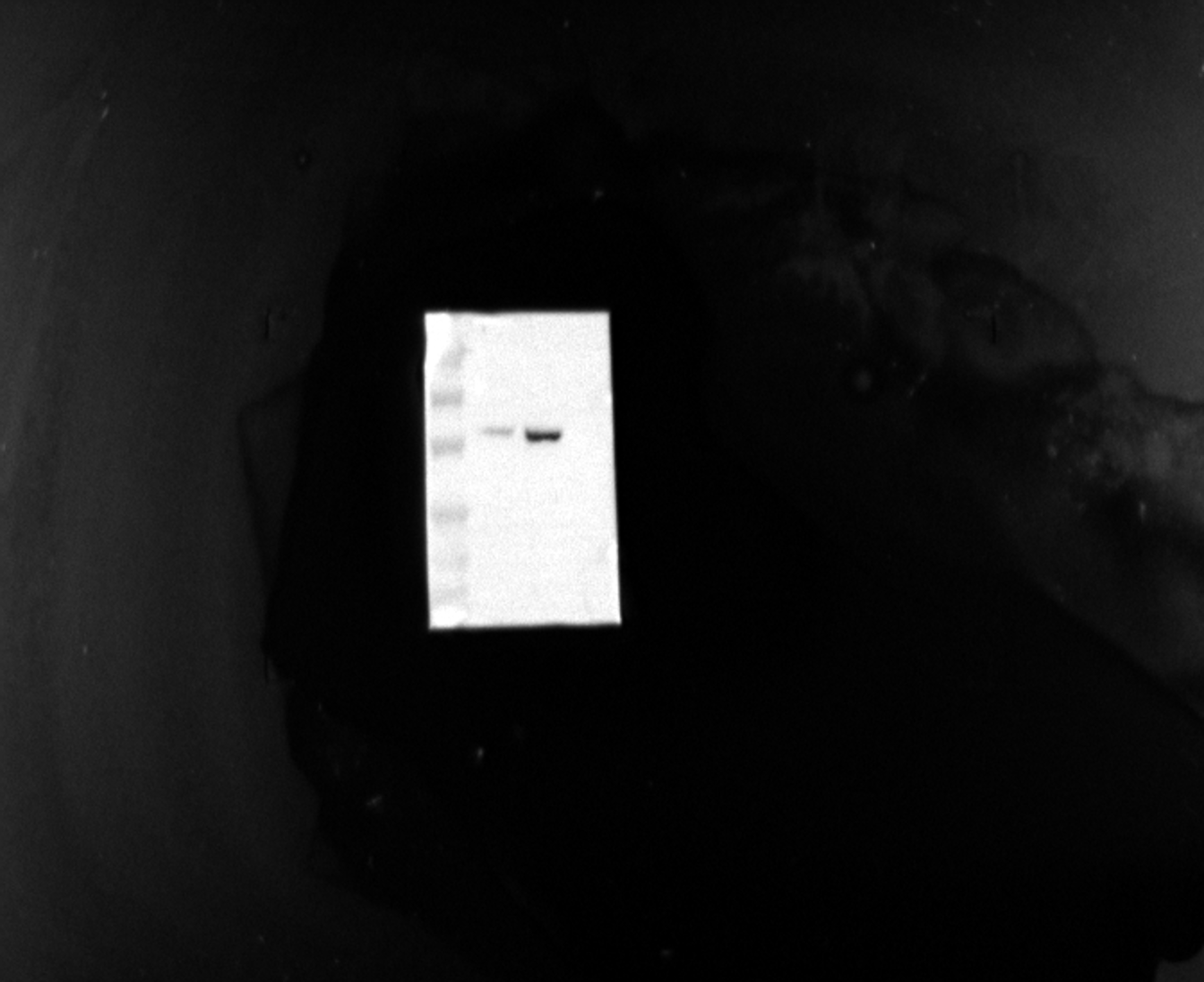
**

Oct4 45kDa

**
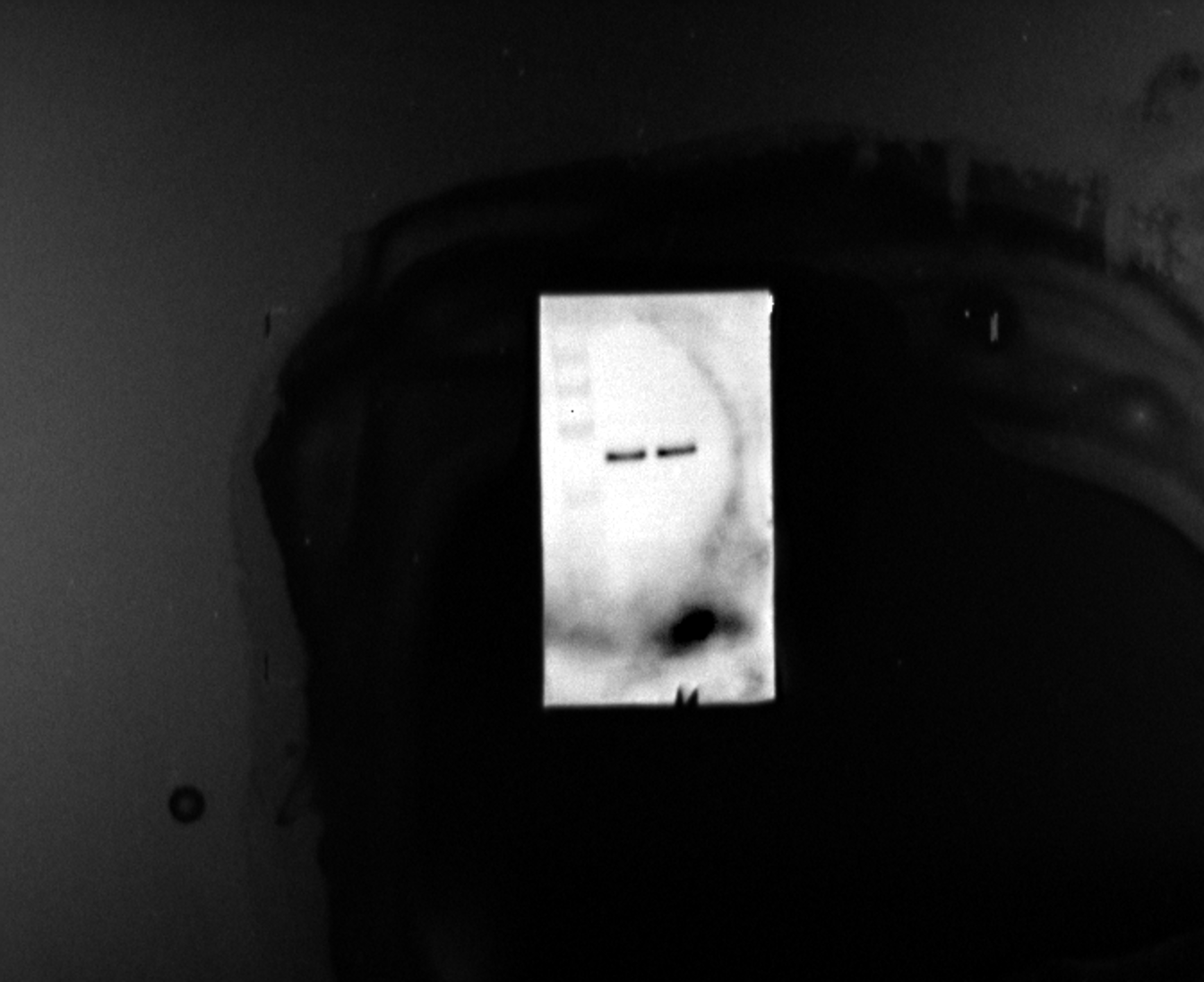
**

GAPDH 36kDa

**Fig.6F CNE2 cell**


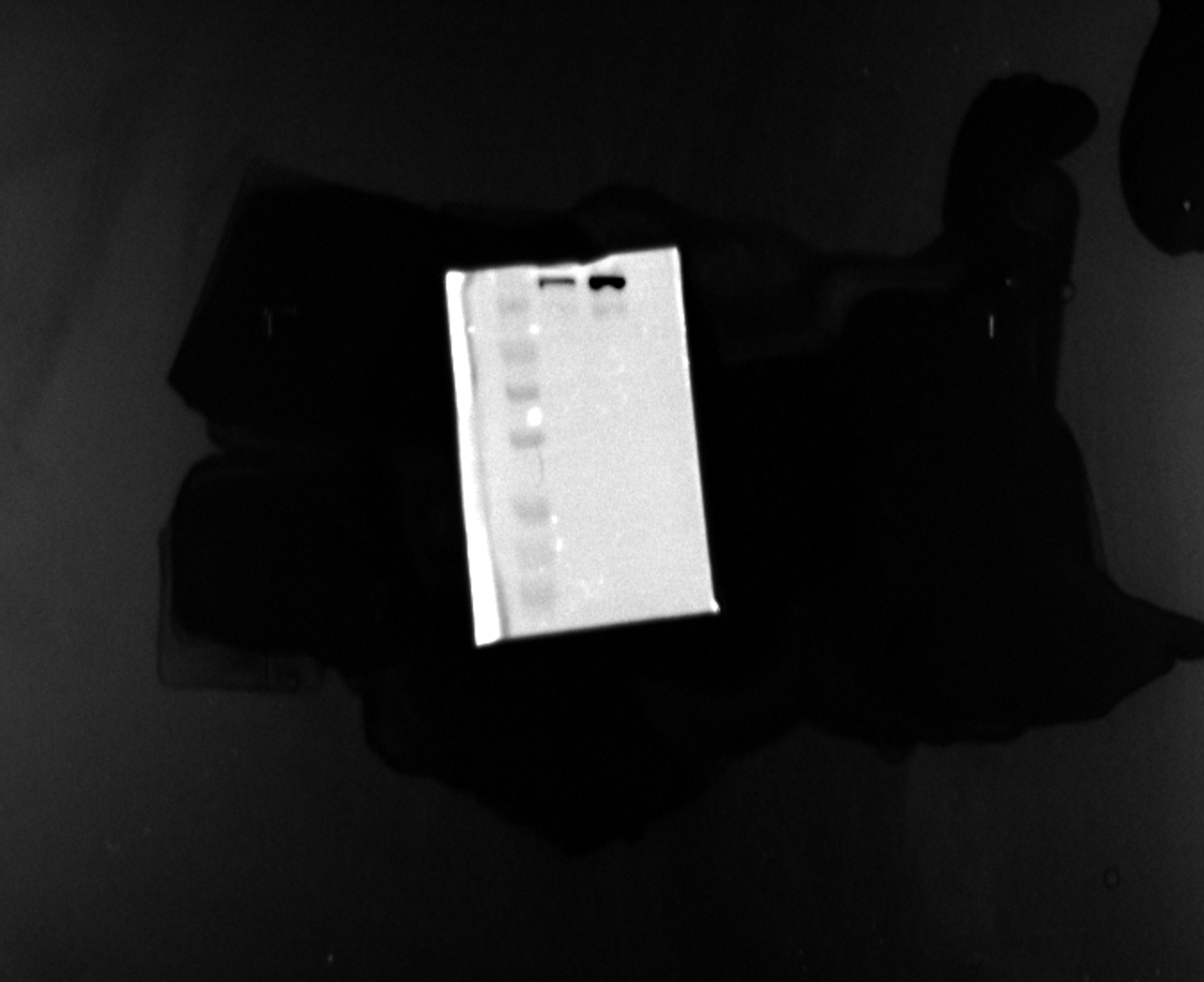


CD133 110 kDa


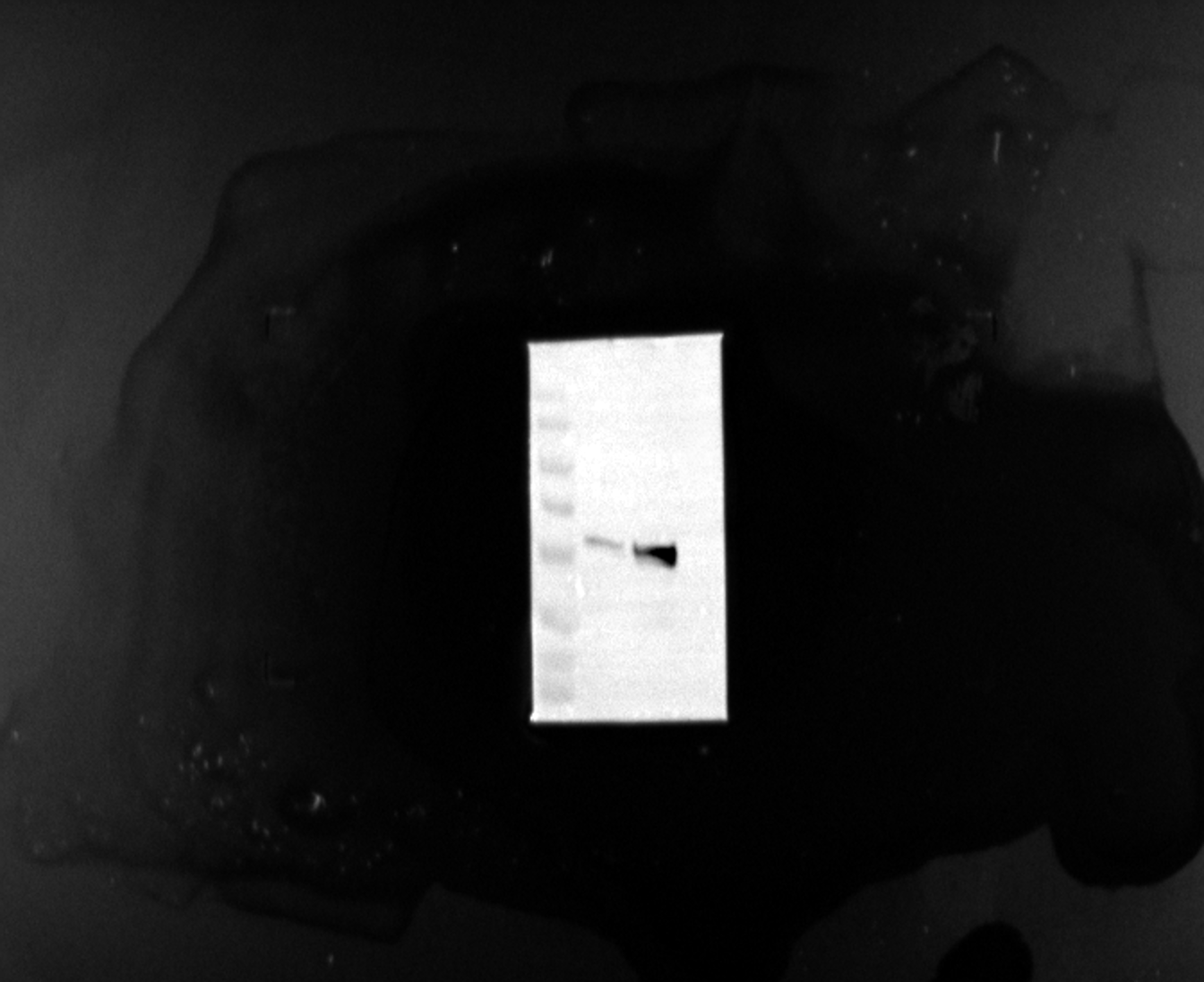


Nanog 42kDa


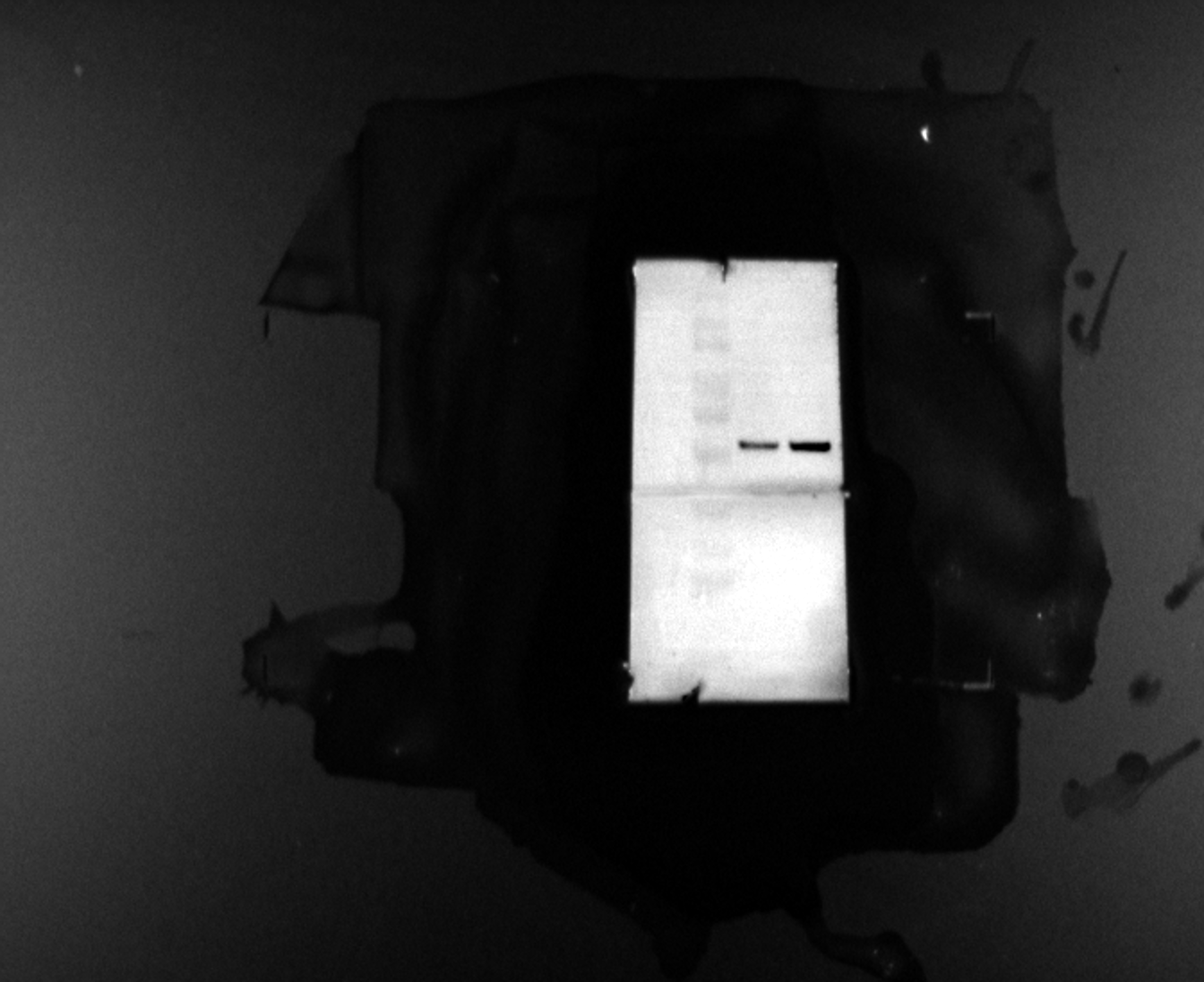


Oct4 45kDa


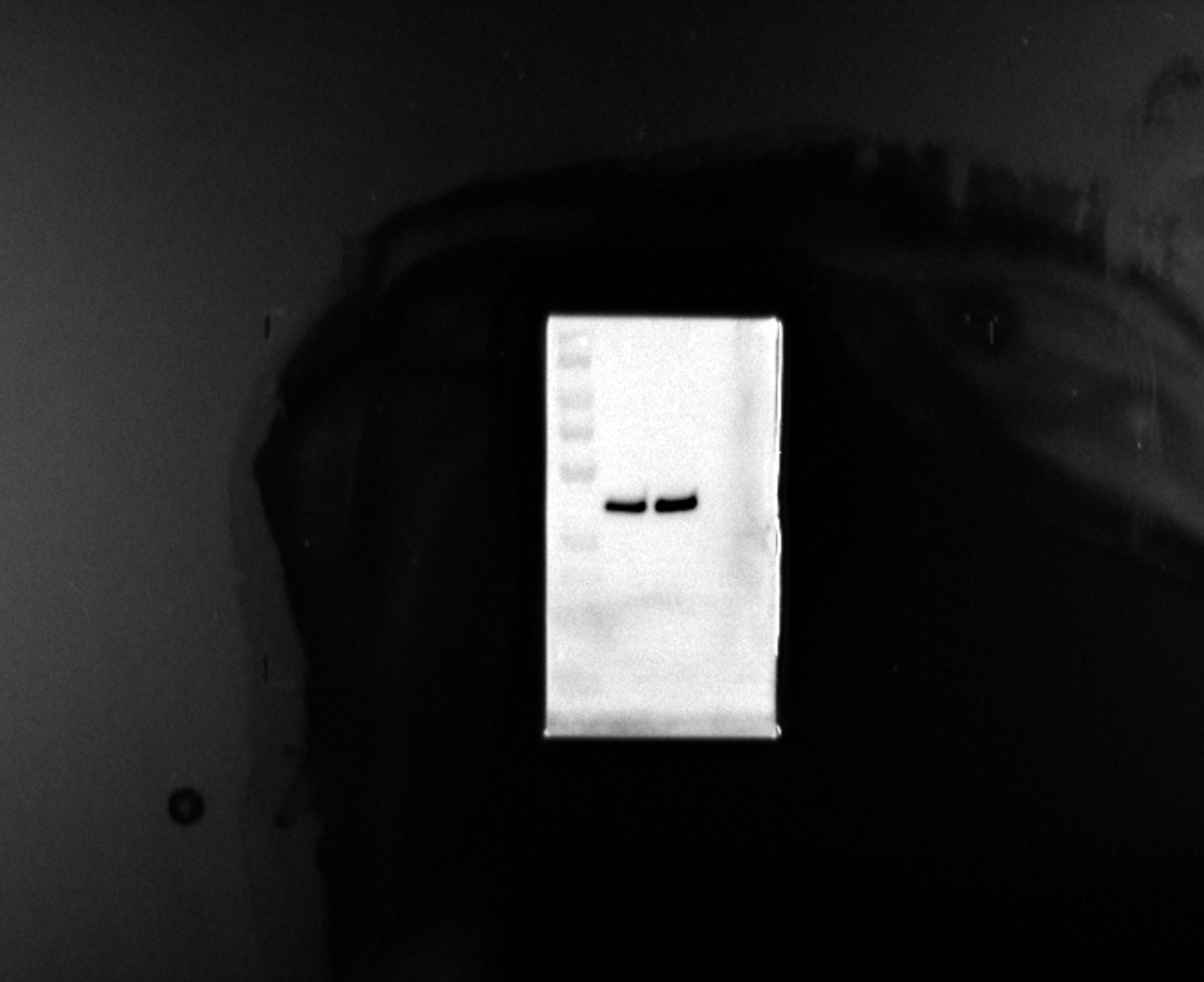


GAPDH 36kDa

N-cadherin
