# Supplementary material for: CircCENPM serves as a CeRNA to aggravate nasopharyngeal carcinoma metastasis and stemness via enhancing BMI1
Source: Hereditas. 2025 Mar 14;162:39. doi: 10.1186/s41065-025-00406-7 (PMC11907939; doi:10.1186/s41065-025-00406-7)
Supplement: Supplementary file 2 — Supplementary Material 2 [file 41065_2025_406_MOESM2_ESM.docx]

**Supplementary materials**

**Supplementary Figure 1**.


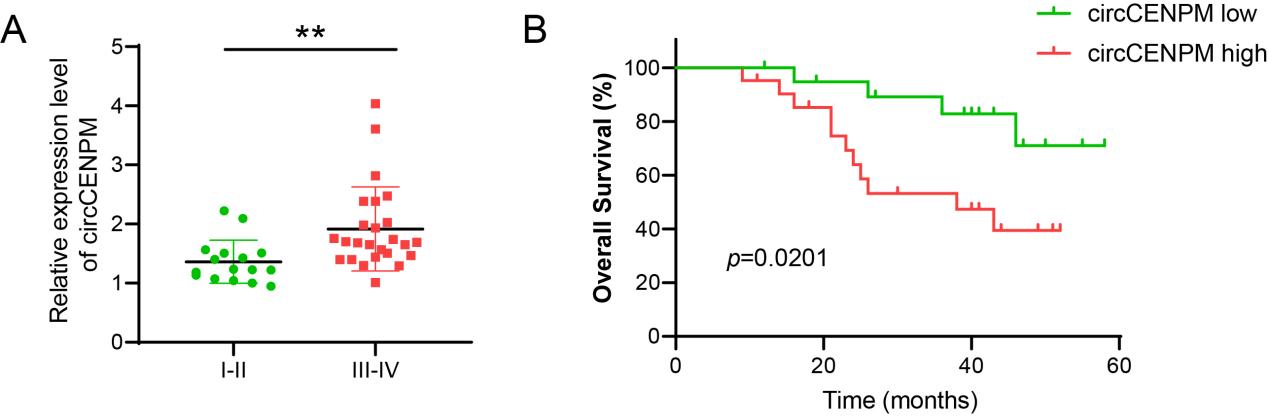


**Figure S1.** The relationship between circCENPM expression and pathological features in NPC patients. A.The expression of circCENPM in 41 NPC tissues of different clinical stages (I+II: 16 cases, III+IV: 25 cases). B. Kaplan-Meier analysis revealed that the expression of circCENPM was a predictive factor of overall survival in NPC (low expression: 20 cases, high expression: 21 cases). ***P* <0.01.
