# Supplementary material for: CircCENPM serves as a CeRNA to aggravate nasopharyngeal carcinoma metastasis and stemness via enhancing BMI1
Source: Hereditas. 2025 Mar 14;162:39. doi: 10.1186/s41065-025-00406-7 (PMC11907939; doi:10.1186/s41065-025-00406-7)
Supplement: Supplementary file 3 — Supplementary Material 3 [file 41065_2025_406_MOESM3_ESM.docx]

**Supplementary Table 1**

**Relationship between circCENPM expression and clinicopathologic features of NPC patients**

| **Characteristics** | **Patients** | **circCENPM** | | ***P*-value** |
| --- | --- | --- | --- | --- |
|  | **n=41** | **low (n=20)** | **high (n=21)** |  |
| **Age (years)** |  |  |  |  |
| ≤50 | 17 | 9 | 8 | 0.6537 |
| >51 | 24 | 11 | 13 |  |
| **Sex** |  |  |  |  |
| Male | 29 | 13 | 16 | 0.4312 |
| Female | 12 | 7 | 5 |  |
| **Smoking status** |  |  |  |  |
| Nonsmokers | 26 | 15 | 11 | 0.1328 |
| Smokers | 15 | 5 | 10 |  |
| **Clinical stage** |  |  |  |  |
| I-II | 16 | 12 | 4 | **0.0072** |
| III-IV | 25 | 8 | 17 |  |
| **Pathological type** |  |  |  |  |
| Nonkeratinizing | 35 | 16 | 19 | 0.3428 |
| Keratinizing | 6 | 4 | 2 |  |
| **Lymphatic metastasis** |  |  |  |  |
| No | 20 | 13 | 7 | **0.0426** |
| Yes | 21 | 7 | 14 |  |
